# Prediction of protein–ligand binding affinity from sequencing data with interpretable machine learning

**DOI:** 10.1038/s41587-022-01307-0

**Published:** 2022-05-23

**Authors:** H. Tomas Rube, Chaitanya Rastogi, Siqian Feng, Judith F. Kribelbauer, Allyson Li, Basheer Becerra, Lucas A. N. Melo, Bach Viet Do, Xiaoting Li, Hammaad H. Adam, Neel H. Shah, Richard S. Mann, Harmen J. Bussemaker

**Affiliations:** 1 Department of Bioengineering, University of California, Merced, Merced, CA USA; 2 Department of Biological Sciences, Columbia University, New York, NY USA; 3 Department of Biochemistry and Molecular Biophysics, Columbia University, New York, NY USA; 4 Department of Chemistry, Columbia University, New York, NY USA; 5 Department of Systems Biology, Columbia University, New York, NY USA

**Keywords:** Kinases, High-throughput screening, Machine learning, DNA methylation, Transcriptional regulatory elements

## Abstract

Protein–ligand interactions are increasingly profiled at high throughput using affinity selection and massively parallel sequencing. However, these assays do not provide the biophysical parameters that most rigorously quantify molecular interactions. Here we describe a flexible machine learning method, called ProBound, that accurately defines sequence recognition in terms of equilibrium binding constants or kinetic rates. This is achieved using a multi-layered maximum-likelihood framework that models both the molecular interactions and the data generation process. We show that ProBound quantifies transcription factor (TF) behavior with models that predict binding affinity over a range exceeding that of previous resources; captures the impact of DNA modifications and conformational flexibility of multi-TF complexes; and infers specificity directly from in vivo data such as ChIP-seq without peak calling. When coupled with an assay called *K*_D_-seq, it determines the absolute affinity of protein–ligand interactions. We also apply ProBound to profile the kinetics of kinase–substrate interactions. ProBound opens new avenues for decoding biological networks and rationally engineering protein–ligand interactions.

## Abstract (web summary)

Protein–ligand binding affinity is predicted quantitatively from sequencing data.

## Main

Critical cellular processes, such as gene regulation and signal transduction, rely on sequence-specific molecular recognition to guide constituent proteins to preferentially interact with specific nucleic acid or polypeptide ligands. The strength and specificity of such ‘sequence recognition’ often spans orders of magnitude, and even weak ligands can be functional^1–3^. Thus, it is essential to comprehensively and quantitatively profile sequence recognition to decode these molecular networks.

Massively parallel sequencing has substantially increased the speed with which sequence recognition can be profiled. In particular, high-throughput methods that couple sequencing with in vitro selection on random ligand pools have emerged as powerful tools for the unbiased profiling of molecular interactions. This includes SELEX methods for TFs^4–14^ and RNA-binding proteins^15,16^ as well as protein display methods for proteases^17^ and T cell receptors^18^. As the randomized ligand pools used in these assays are extremely complex (and most sequences are observed rarely, if ever), machine learning methods have become essential for synthesizing sequencing data into ‘recognition models’ that encode how any sequence is recognized.

In recent years, several methods—using deep learning^19–21^, probabilistic mixture models^22^ or high-dimensional embedding^23^—have been developed to analyze TF:DNA binding data. However, although protein interactions are most rigorously quantified in terms of biophysical parameters such as dissociation constants (*K*_D_), most of these methods focus on classifying sequences as bound or free or assign non-biophysical binding scores. Although some biophysical methods have been developed^24,25^, they are limited to estimating relative *K*_D_ values for TFs and cannot systematically model SELEX enrichment over multiple rounds. Furthermore, although new assays have been developed to profile in vivo effects beyond direct sequence recognition^9,12,13,26^, no current computational method can synthesize such complementary experiments into a unified binding model that captures the impact of co-factors and DNA methylation.

In this study, we solve these problems with a flexible machine learning framework, called ProBound, which is capable of learning biophysically interpretable models by synthesizing a wide range of sequencing data. Although we set out to analyze multi-round SELEX data, we soon realized that ProBound enabled the development of sequencing assays that probe previously inaccessible biophysical parameters. To illustrate this, we introduce *K*_D_-seq (which measures absolute *K*_*D*_ values using the input, bound and unbound SELEX fractions) and Kinase-seq (which profiles kinase substrate specificity using a multi-time-point protein display assay). More broadly, our results illustrate how classical biochemical assays, which often use multiple fractions, time points or concentrations, can be upgraded with sequencing and principled machine learning to conduct biophysical measurements at unprecedented scale.

## ProBound framework

**Fig. 1:**
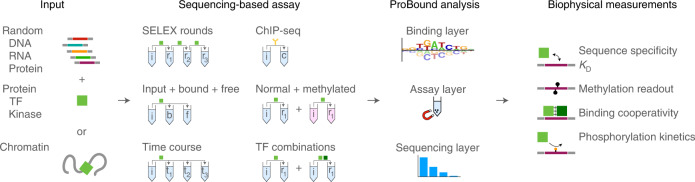
Overview of the ProBound method. A wide range of high-throughput experiments use selection on libraries of DNA, RNA or displayed protein molecules coupled with sequencing to characterize sequence-specific molecular interactions. ProBound uses machine learning tailored to model the recognition, selection and sequencing processes underlying these data to infer biophysically meaningful recognition models.

ProBound uses three layers to systematically model multi-library sequencing data (Fig. 1 and Methods): a binding layer predicts the binding free energy or enzymatic efficiency from sequence using a sequence recognition model; an assay layer encodes the selection steps that generated the libraries and predicts frequencies of all ligands; and a sequencing layer models the stochastic sampling of the libraries during sequencing. These layers are combined in a likelihood function, which is optimized to infer the recognition model. Although many ligands have noisy counts or are entirely missing due to the complexity of randomized libraries, the final recognition model is robust because it has to optimally explain the full sequencing dataset. Each layer is easily extensible; for example, the binding layer, which, by default, corresponds to a position-specific affinity matrix^27^, can be extended to include base–base interactions or cooperative binding by multiple TFs. Flexibility in the assay layer enables the modeling of alternative processes, such as enzymatic modification. Finally, multiple assays can be analyzed jointly to profile more complex phenomena (for example, methylation sensitivity).

## A compendium of accurate TF binding models

**Fig. 2:**
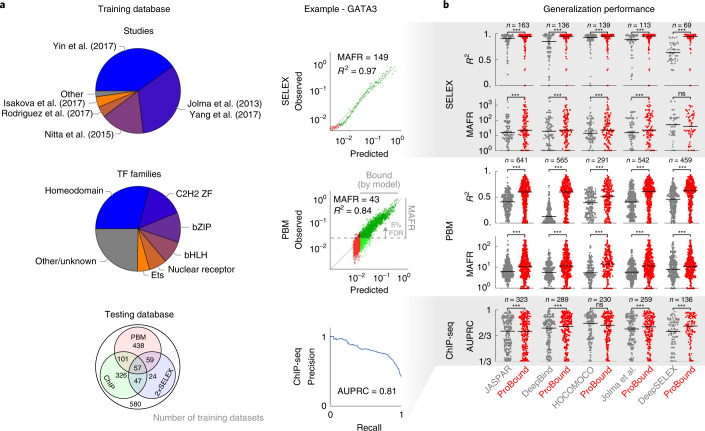
Validation of TF binding model performance. **a**, Breakdown of the training dataset used to build binding models by originating study and TF family (pie charts) and by availability of testing data used to evaluate them (Venn diagram). Representative SELEX (top) and PBM (middle) comparisons of observed and model-predicted binding signals used to quantify generalization performance. Each point in the scatterplots corresponds to either 500 SELEX probes or ten PBM probes; green indicates where the model predicts binding above an estimated baseline (Methods), whereas darker points indicate the MAFR of observed binding signal over which, at most, 5% of predicted binding was below the baseline. Representative precision-recall curve (bottom) for the ChIP-seq peak classification task used to quantify model performance in terms of AUPRC (1/3 corresponds to a random classifier). **b**, Performance comparison of ProBound models versus popular existing resources. For each ProBound and resource model pair (points), the average score was computed for all matching testing datasets. Horizontal bars indicate median performance. Significance was computed using the two-sided Wilcoxon signed-rank test (*** indicates *P* < 10^−3^).

Our initial objective was to analyze thousands of published SELEX datasets^7,8,10,12,13,28–30^ and produce high-quality TF binding models that capture low-affinity binding, an important yet difficult-to-detect gene regulatory phenomenon^1–3,25^. This required us to quantify TF sequence recognition over a wide affinity range rather than merely classify sequences as ‘bound’ or ‘unbound’. We, therefore, assembled a training database of published SELEX experiments, which we analyzed with a uniform computational pipeline, yielding 1,632 binding models (Fig. 2a, Supplementary Table 1 and Methods). To assess the generalization performance of our models, we linked each TF to published protein-binding microarray (PBM), chromatin immunoprecipitation with sequencing (ChIP-seq) and non-training SELEX data. We computed three complementary performance metrics: meaningful affinity fold range (MAFR), a metric that provides a conservative bound on the ability of a model to detect low-affinity binding; *R*^2^, the fraction of signal variance explained by the model; and area under the precision-recall curve (AUPRC), a common metric^19,20,25,31^ for quantifying how well a model classifies genomic regions as bound or unbound as determined by ChIP-seq peaks^32^. We used these to benchmark our models to those in major resources and surveys, linking all JASPAR^33^, DeepBind^19^, HOCOMOCO^34^, Jolma et al.^28^ and recently published DeepSELEX^20^ models by TF. On average, ProBound outperformed these resources across all metrics (Fig. 2b), with the PBM and SELEX metrics displaying the largest improvement. Two comparisons—HOCOMOCO ChIP-seq AUPRC and DeepBind SELEX *R*^2^—showed no significant difference. The less notable improvement in AUPRC is likely due to bias toward high-affinity sequences in ChIP-seq peaks, for which accurate low-affinity predictions are less relevant^25^. Below, we will introduce an alternative method for analyzing ChIP-seq data that eliminates the need for ChIP-seq peak discovery.

**Extended Data Fig. 1:**
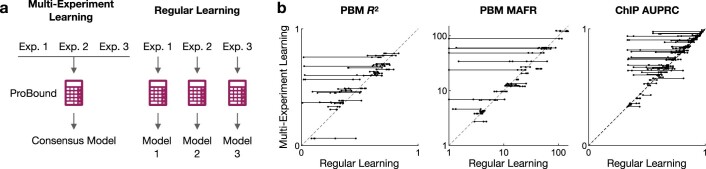
Integrative analysis of multiple TF SELEX datasets produces consensus binding models. (a) Schematic contrasting ProBound’s multi-experiment learning strategy that builds a consensus model for a TF by simultaneously training on all relevant SELEX data for the TF with the traditional approach that builds independent models for every individual dataset. (b) Generalization performance of consensus binding models (y-axis) and single-experiment models (x-axis) on three different metrics (scatterplots). Points correspond to models trained on individual experiments and lines connect experiments used to build the corresponding consensus model. Points above the diagonal correspond to instances where the consensus model outperforms single-experiment models.

Over the years, several TFs have been assayed many times by different research groups and SELEX platforms. We reasoned that jointly analyzing such data would produce a ‘consensus’ model focused on the true binding signal rather than platform-specific biases (Extended Data Fig. 1a). Such consensus models displayed significantly improved performance when compared to traditional single-experiment models (Extended Data Fig. 1b), indicating that multi-experiment analysis can improve binding predictions.

To facilitate adoption by other researchers, we have made a curated version of our models, comparative analyses and computational tools readily available through a comprehensive resource at motifcentral.org.

## Quantifying TF binding cooperativity

Variables beyond sequence, such as co-factor interactions and DNA methylation, substantially influence TF behavior in vivo, and, therefore, TF binding models must account for them to improve binding predictions. We first focused on co-factors, which modulate TF binding in a cell-type-specific manner. Despite the growing number of SELEX assays characterizing TF complexes^7,9,26^, it remains a challenge to quantify sequence recognition in a way that clearly separates the contributions from many potential TF complexes and their various internal structural configurations—a problem that grows exponentially with the number of factors assayed. In an approach that builds upon our multi-experiment framework, we measure subunit binding specificity and cooperativity by explicitly modeling the allowed complexes in multiple SELEX datasets that probe different TF combinations.

**Fig. 3:**
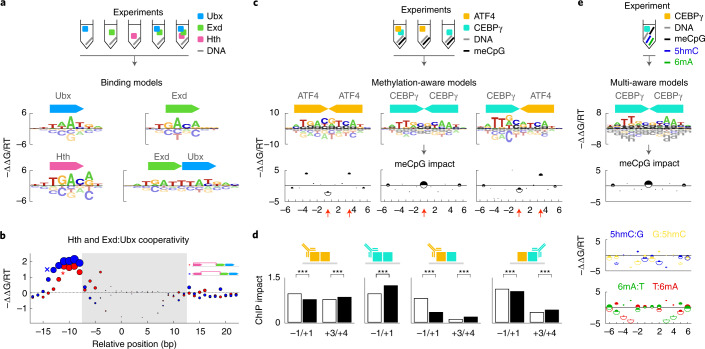
Integrated modeling of complementary assays quantifies the impact of methylation and co-factors on TF binding. **a**, Combinations of TFs assayed (top) and unified model learned by ProBound (bottom). The model consists of the inferred energy logos for the monomeric and dimeric complexes (motifs) and the (**b**) inferred binding cooperativity (*y* axis) between Hth and Exd:Ubx for different relative positions (*x* axis) and orientations (red: parallel; blue: anti-parallel) of the subunits. Disk areas proportional to the affinity of the strongest predicted sequence highlight the most stable configurations. The shaded region indicates overlapping motifs. Schematics (inset) illustrate two configurations indicated on the plot. **c**, Combinations of TFs and methylated/unmethylated libraries assayed (schematic); methylation-aware binding models (motifs) using the alphabet in Extended Data Fig. 4a; and the impact of meCpG on binding free-energy (plots; −ΔΔ*G*_CpG→meCpG_/RT on *y* axis) as a function of position within the binding site (*x* axis). Half-disk areas are proportional to the maximum affinity when either CpG (white) or meCpG (black) is substituted at the corresponding position in the highest-affinity sequence and highlight positions with high-affinity methylation readout. **d**, Impact of substituting a CpG (white) or meCpG (black) at a specific position in the highest-affinity binding site as quantified using ChIP-seq data. Each pair of bars corresponds to a substitution at a specific position and to red arrows in **c**. Antibody symbols indicate respective immunoprecipitated factor. *P* values were computed using an *F*-test (one-sided, *** indicates *P* < 10^−3^; Methods and Supplementary Table 2). **e**, Same as **c** for data simultaneously measuring methylation readout for meCpG, 5hmC and 6mA modifications.

**Extended Data Fig. 2:**
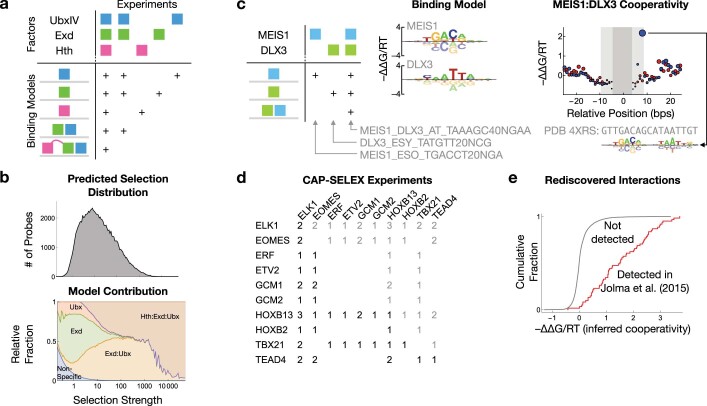
Integrative modeling to quantify TF binding cooperativity. (a) Schematic table describing the combinations of TFs assayed in five experiments (top) that were jointly analyzed to produce binding models of the different monomers and their complexes (bottom) by explicitly defining which models can form in each experiment (+ sign). (b) Distribution of probes (top) and the predicted relative contribution of every recognition mode (bottom) as a function of predicted binding selection strength (x-axis) in the first round of selection from SELEX-seq data assaying Hth, Exd, and UbxIV. (c) Integrative modeling of HT-SELEX and CAP-SELEX data for MEIS1 and DLX3 (schematic table) yields binding models for the monomers (energy logos) and configuration-dependent binding cooperativity for the MEIS1:DLX3 complex (same circle plot representation as in Fig. 3b). The bottom right logo shows the specificity of MEIS1:DLX3 for the most stable configuration (connecting arrow), aligned to a sequence previously crystallized with MEIS1:DLX31. (d) Table showing the availability of CAP-SELEX data for different TF-TF combinations. The 10 TFs with the most identified co-factors are included, and numbers indicate replicate count. (e) Distribution plot comparing the binding cooperativity inferred by ProBound at the configurations that were identified as cooperative in the original CAP-SELEX study (red line) and at all other configurations (gray line). The models were trained on the CAP-SELEX data tabulated in (d) and are shown in Extended Data Figure 3.

We first applied this method on the complex formed by three highly conserved *Drosophila* homeodomain proteins: Homothorax (Hth), Extradenticle (Exd) and Ultrabithorax (Ubx). Previous studies showed that Ubx and Exd form fixed-spacer heterodimers^8,25^ and that Hth uses multiple relative spacings to bind cooperatively with similar heterodimers^26^. To characterize Hth:Exd:Ubx, we first performed SELEX-seq with all three factors and then analyzed these data in conjunction with our previous monomer and heterodimer data (Fig. 3a and Extended Data Fig. 2a). We modeled the ternary complex with two subunits representing Hth and Exd:Ubx; the total binding energy was the sum of their independent binding specificities and of a cooperativity term that depended on their relative position and orientation.

The resulting model revealed substantial cooperativity (ΔΔ*G*_config_ ≈ 2RT) when Hth binds 8–13 base pairs (bp) upstream of Exd:Ubx (Fig. 3b), which, along with our monomer and heterodimer models, mirrored previous results^25,26^. Although a larger spacing is tolerated when Hth is reversed, cooperativity is lost when Hth binds far away from the Exd:Ubx half-site, regardless of orientation. As expected, selection in the Hth-Exd-Ubx experiment was driven by multiple subcomplexes (Extended Data Fig. 2b), underscoring the need to simultaneously model all preferences.

**Extended Data Fig. 3:**
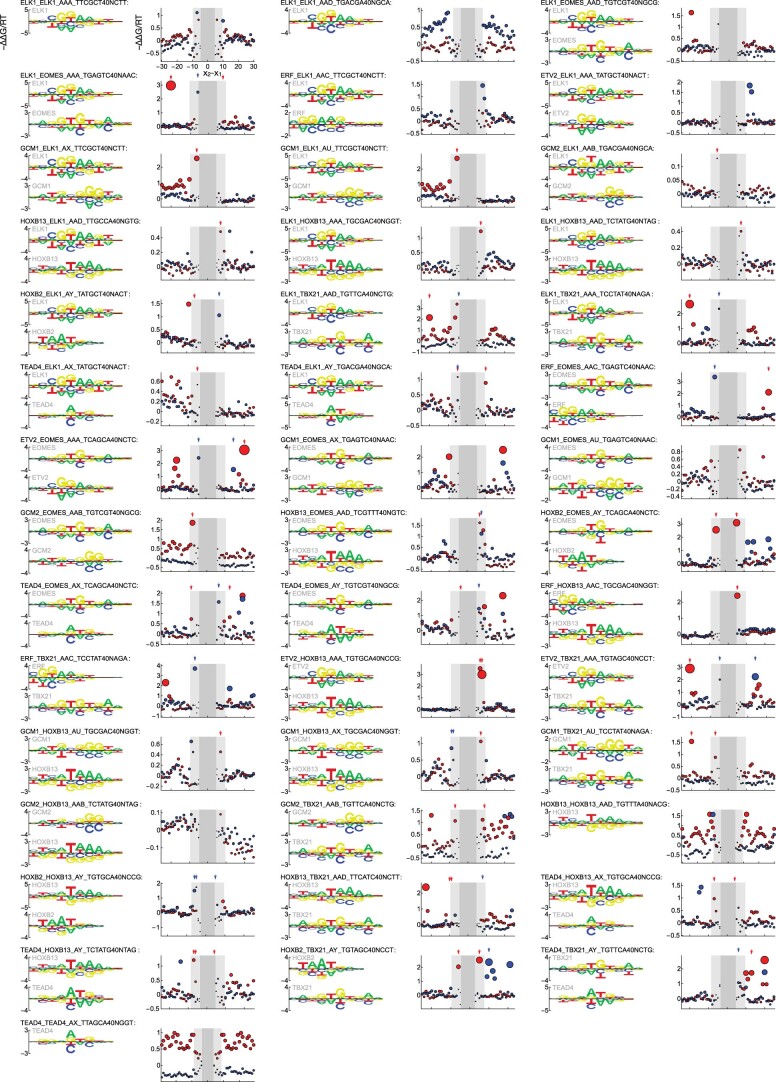
Binding models learned through joint analysis of CAP-SELEX and HT-SELEX data. Models are displayed as in Extended Data Figure 2c. Red and blue arrows indicate the configurations identified as cooperative in the original analysis of each dataset. These configurations (which correspond to the red line in Extended Data Figure 2e) were identified by aligning the inferred monomer binding modes to the position-probability matrices reported in the original study and selecting the configuration that minimizes the KL divergence.

To further validate our approach, we reanalyzed published data^9^ for the human TF heterodimer MEIS1:DLX3 and found strong cooperativity at the exact same configuration (i.e., relative spacing and orientation) previously confirmed^9^ using X-ray crystallography (Extended Data Fig. 2c). Subsequent systematic analysis of data for all pairwise combinations of the top ten most interacting TFs from the same study (Extended Data Fig. 2d) produced binding models with significant cooperativity for previously reported^9^ configurations (Extended Data Fig. 2e; *P* = 1.5 × 10^−30^, Mann–Whitney test) and provided evidence of cooperativity for many other ones as well (Extended Data Fig. 3).

## Learning methylation-aware TF binding models

**Extended Data Fig. 4:**
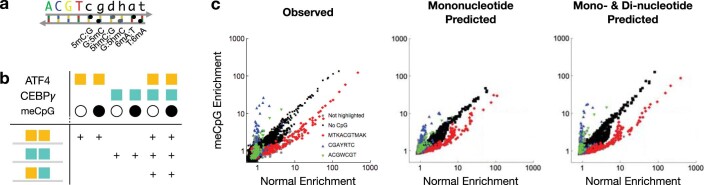
Learning methylation-aware binding models from EpiSELEX-seq data. (a) Alphabet used to represent normal and methylated base pairs. (b) Same as Extended Data Figure 2a, but showing the combinations of ATF4, CEBP*γ*, and normal and methylated DNA that were included in each experiment and the resulting complexes that were modeled. (c) K-mer enrichment analysis for the observed ATF4 EpiSELEX-seq read counts (left), the counts predicted by a mononucleotide-only model (middle), and the counts predicted by a mono- and di-nucleotide model (right). Each scatterplot compares the 8-mer enrichment observed in the normal (x-axis) and methylated (y-axis) libraries. Every point represents an 8-mer and is colored according to the legend; color is assigned based on a 6bp matching substring between the 8mer and the IUPAC code.

Next, we focused on another variable affecting in vivo binding: DNA methylation. Chemical modifications to DNA, such as fully methylated CpG dinucleotides (meCpG), are common epigenetic marks that can alter TF binding and, thus, gene regulation^35–38^. Unlike existing methods that compare methylated and normal SELEX libraries to detect TF ‘methylation readout’ at the level of enriched subsequences^12,14,39^, we used ProBound with an extended alphabet (Extended Data Fig. 4a and Methods) and our multi-experiment framework to learn methylation-aware binding models that resolve the position-specific impact of methylation (ΔΔ*G*_CpG→meCpG_), enabling binding predictions for any (un)methylated sequence.

We tested this approach by analyzing the effect of meCpG on the ATF4:CEBP*γ* heterodimer while controlling for the confounding influence of the respective homodimers. Using data for all combinations of ATF4/CEBP*γ* and normal/methylated DNA (Extended Data Fig. 4b), we simultaneously learned methylation-aware binding models for all three dimers (Fig. 3c and Methods). These predict methylation-induced stabilization/destabilization patterns (Fig. 3c and Extended Data Fig. 4c) consistent with previous analyses of the ATF4 homodimer^13^ and similar to those of the related CEBP*β* homodimer^13^ and ATF4:CEBP*β* heterodimer^39^. Strikingly, ATF4 overrides CEBP*γ* to retain its methylation readout at the central position of the heterodimer complex. We used ChIP-seq data to estimate the impact of these position-specific methylation sensitivities in vivo and found that methylation significantly affected binding in the direction predicted by our models (Fig. 3d and Methods).

**Extended Data Fig. 5:**
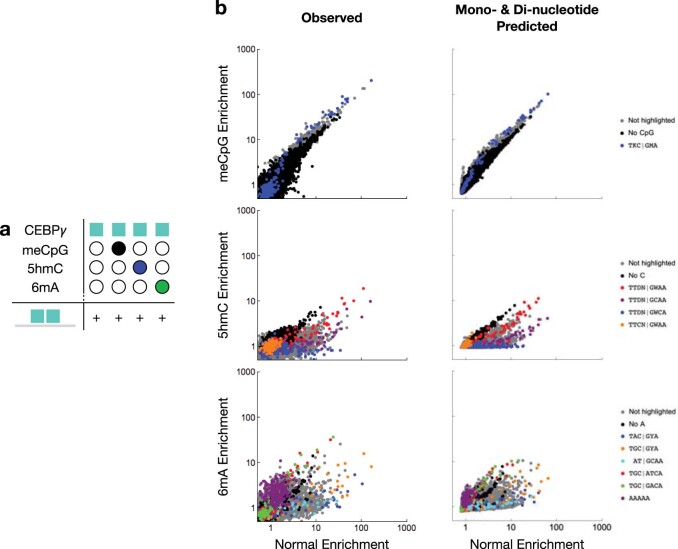
Extending EpiSELEX-seq to measure the impact of 5hmC and 6mA on CEBP*γ* binding. (a) Schematic table describing the factors, library and binding model used in analyzing the extended EpiSELEX-seq assay (cf. Extended Data Figure 4b). (b) K-mer enrichment analysis comparing normal and modified EpiSELEX-seq libraries, computed and displayed as in Extended Data Figure 4c.

Other DNA modifications, such as *N*^6^-methyladenine (6mA) and 5-hydroxymethylcytosine (5hmC), can also be functional^40–45^. To characterize their impact on TF binding, we extended the EpiSELEX-seq protocol to assay multiple sub-libraries simultaneously: unmethylated, meCpG, 5hmC and 6mA (Fig. 3e and Extended Data Fig. 5a). Not only is this simpler than assaying each methylation mark separately, it also reduces experimental error. Repeating the binding assay for CEBP*γ* and jointly analyzing all four libraries revealed substantial and distinct stabilization/destabilization patterns for both 5hmC and 6mA (Fig. 3e and Extended Data Fig. 5b). Notably, the inferred meCpG methylation sensitivity is identical to what we found above. These results illustrate both the versatility of our approach and the fact that 5hmC and 6mA can have a substantial impact on binding.

## Measuring absolute binding constants using SELEX

**Fig. 4:**
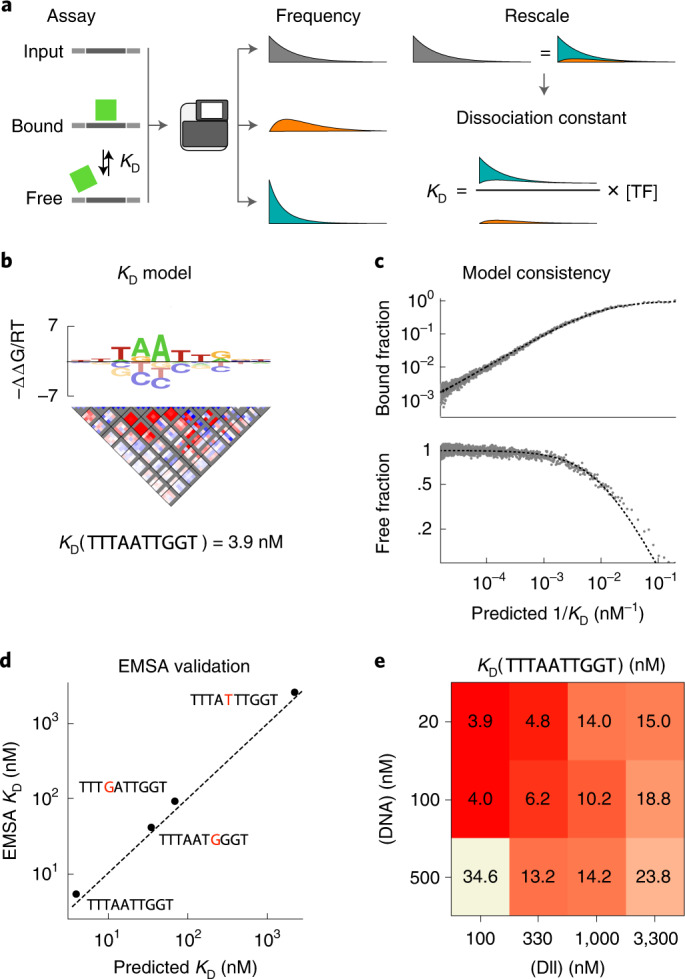
ProBound infers absolute *K*_D_ values. **a**, Schematic overview of the *K*_D_-seq method. After a TF is incubated with a randomized DNA library, the bound, free and input probes are sequenced, measuring the relative probe frequencies in each fraction. This can be used to estimate the absolute binding probabilities (and, hence, *K*_D_) with a sum rule that relates the three frequencies. **b**, *K*_D_ model for Dll consisting of a specificity model with an energy logo (top) and an interaction matrix (middle), which together predict the relative binding affinity, and the absolute *K*_D_ for a reference sequence (bottom). The interaction plot shows stabilizing (red) and destabilizing (blue) corrections to the energy logo for each pair of positions (boxes) and bases (pixels) in the logo. Gray indicates prohibited corrections. Model generated from data where [Dll] = 100 nM and [DNA] = 20 nM. **c**, Comparison of the predicted ${K}_{{{{\rm{D}}}}}^{-1}$ (*x* axis) and observed probe fractions (*y* axis) in the bound (top) and free (bottom) libraries. Points represent the average observed fraction for 500 probes binned by predicted *K*_D_. The dashed line indicates expected value assuming equilibrium binding model. **d**, Comparison between EMSA-measured (*y* axis) and model-predicted (*x* axis) *K*_D_ values for four probes. The dashed line indicates perfect agreement. **e**, *K*_D_ of the sequence TTTAATTGGT as estimated by *K*_D_-seq for different Dll and DNA concentrations.

Although we have focused on quantifying binding specificity in terms of relative affinities, knowledge of absolute affinities is necessary for predicting equilibrium occupancy and for comparing different TFs on a common scale. Fundamentally, SELEX assays probe relative ligand frequencies and, so far, have only been used to estimate relative affinities. To overcome this limitation, we developed an assay called *K*_D_-seq. It uses ProBound to jointly analyze the input, bound and free probes from a selection round to produce both a specificity model and an estimate of the absolute dissociation constant (*K*_D_) for a reference sequence. Intuitively, *K*_D_-seq uses a sum rule that relates the relative ligand frequencies of the three libraries to infer absolute binding probabilities, which are then converted to *K*_D_ estimates in a way that corrects for binding saturation (Fig. 4a and Methods).

**Extended Data Fig. 6:**
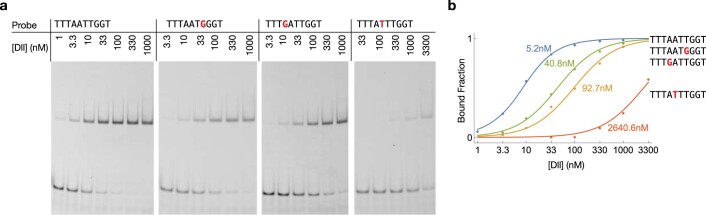
EMSA validation measurements. (a) EMSA experiments for Dll and four DNA probes. (b) Fraction bound DNA probes predicted by the equilibrium binding model (lines, computed using indicated K_D_ values and equation (45)) and estimated based on EMSA band intensities (dots).

**Extended Data Fig. 7:**
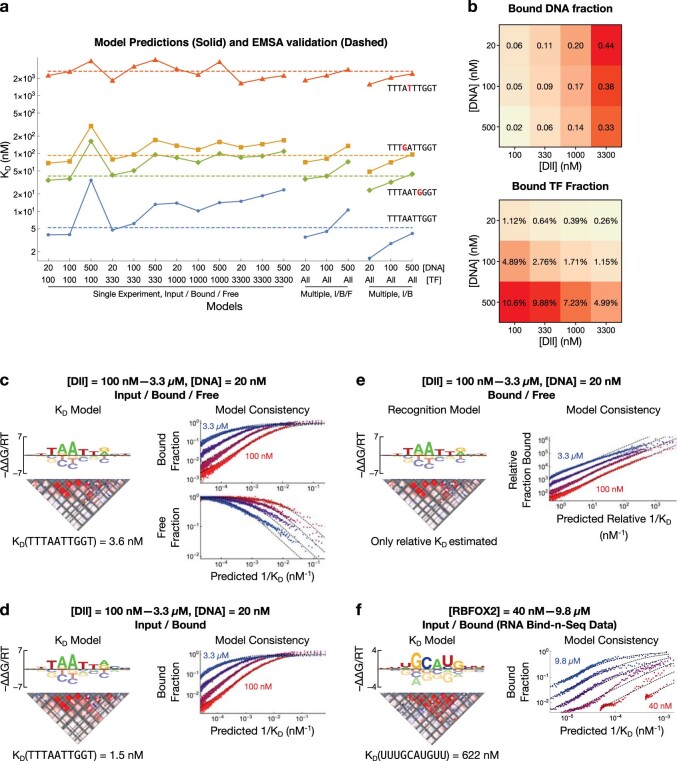
The robustness of *K*_*D*_-seq. (a) Comparison between EMSA-measured (dashed line) and different model-predicted (points) K_D_ values for four binding probes. Various model training strategies (x-axis) used different sequencing libraries: the input/bound/free libraries from a single experiment (left); the input/bound/free libraries from multiple experiments at different TF concentrations (center); or the input/bound libraries from multiple experiments at different TF concentrations (right). (b) Fraction of DNA bound (top) and fraction of TF bound (bottom) as inferred by ProBound when learning binding models from individual K_D_-seq experiments (cf. left points in (a)). (c) Example K_D_ model (left) and observed and predicted probe enrichments (right; cf. Fig. 4c) for a model from the central points in (a). (d) Same as (c), but for a model from the right points in (a). (e) Same as (c), but only using the bound/free libraries (analogous to Spec-seq). This model can only predict relative K_D_, as the bound/free ratio is proportional to K_D_ for all TF concentrations. In addition, the model predicts enrichment in the data up to a global rescaling factor. (f) Same as (d), but for a model derived from RNA Bind-n-Seq data for RBFOX2.

We initially tested *K*_D_-seq using the *Drosophila* homeodomain protein Distal-less (Dll) at low DNA and TF concentrations (100 nM and 20 nM, respectively) to achieve strong enrichment and avoid excessive binding saturation. The resulting model (Fig. 4b) accurately predicted enrichment in the bound and free libraries over three orders of magnitude in *K*_D_ (Fig. 4c). For validation, we measured the *K*_D_ values of the optimal model-predicted binding site and three suboptimal sequences using standard electromobility shift assays and found excellent quantitative agreement (Fig. 4d and Extended Data Fig. 6). We then confirmed the robustness of *K*_*D*_-seq affinity measurements by repeating the assay at different TF and DNA concentrations (Extended Data Fig. 7a). The resulting specificity models were virtually identical (pairwise *r*^2^ for ΔΔ*G* ranging from 0.974 to 0.998), with the fraction of TF and DNA bound changing as expected (Extended Data Fig. 7b). Although the *K*_D_ estimate for the highest-affinity sequence was similar across several conditions, it shifted when the TF concentration was extremely high compared to the *K*_D_ or when the DNA concentration was much higher than that of the TF (Fig. 4e; see ‘Practical guidelines’ in the Methods).

**Extended Data Fig. 8:**
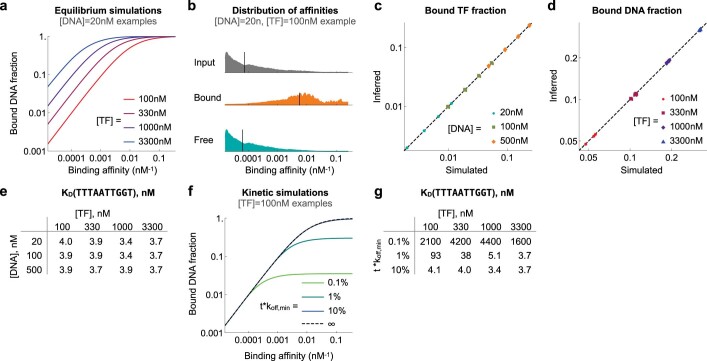
Testing theoretical validity of *K*_D_-seq using equilibrium and kinetic simulations. (a) Plot showing bound fraction vs. binding affinity in simulation of equilibrium binding. ‘Ground truth’ binding affinities were computed using the binding model in Fig. 4b (K_D_ = 3.9nM). Lines correspond to simulations at different total TF concentrations. (b) Distributions of binding affinities in the input, bound and free libraries. Vertical lines indicate the median affinity in each library. (c) Comparison of the bound TF fraction in the simulation (‘truth’) vs. the fraction inferred by ProBound after analyzing the resulting synthetic reads. Each dot corresponds to a simulation with a unique [Dll]/[TF] combination, colored by the DNA concentration. (d) Same as (c) but showing the net bound DNA fraction colored by TF concentration. (e) K_D_ value for the highest-affinity sequence inferred from the synthetic data. (f) Same as (a) but showing the fraction of DNA bound in kinetic simulations using different incubation times t. k_off,min_ is the off-rate for the highest-affinity probe. (g) K_D_ value for the highest-affinity sequence inferred using synthetic data from the kinetic simulations.

To test the theoretical validity of *K*_D_-seq, we used the binding model of Fig. 4b as the ‘ground truth’ and simulated data for a range of Dll and DNA concentrations. In all cases, ProBound accurately recovered the *K*_D_ model (Extended Data Fig. 8a–e). In simulations at various incubation times, ProBound inferred correct *K*_D_ values at times exceeding ~10% of the equilibration time of the slowest probe in the library (Extended Data Fig. 8f,g). Taken together, this shows that *K*_D_-seq is theoretically valid and robust.

ProBound can also learn *K*_D_ models by jointly analyzing the bound and input libraries of multiple SELEX experiments at different TF concentrations. Intuitively, this approach uses saturation effects to determine the absolute affinity scale. For Dll, the *K*_D_ models from the two approaches are very similar (Extended Data Fig. 7a,c,d). When applied to multi-concentration RNA Bind-N-seq^16^ data for RBFOX2, the resulting *K*_D_ model correctly captured the observed transition from linear to saturated selection in the experiments (Extended Data Fig. 7f). Finally, we note that ProBound can estimate relative affinities using only the free and bound libraries, as in the Spec-seq^46^ assay (Extended Data Fig. 7e).

## Peak-free motif discovery from ChIP-seq data

Although the preceding analyses have focused on quantifying the impact of co-factors and TF concentration on in vitro binding, we also wanted to learn their in vivo impact directly from ChIP-seq data. Standard motif discovery algorithms aim to discover overrepresented sequences within discrete genomic regions—identified by ‘peak callers’—that harbor a statistically significant enrichment of ChIP-seq reads. Peak calling is useful for identifying the most prominent genomic binding sites, but it ignores information about *cis*-regulatory logic contained within more weakly bound regions. We hypothesized that ProBound can extract such logic by directly modeling how the input and ChIP libraries relate to each other.

**Fig. 5:**
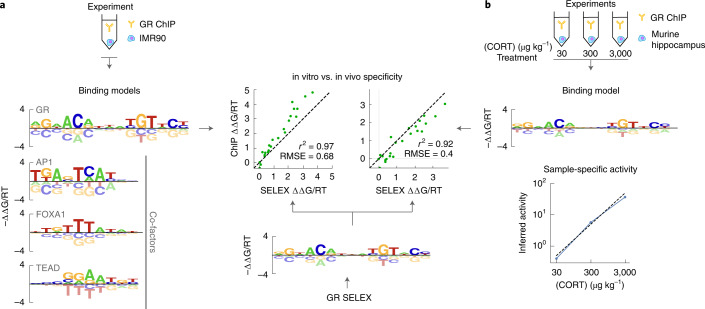
ProBound learns quantitative binding models and sample-specific TF activities using peak-free ChIP-seq analysis. **a**, Binding models for GR and three co-factors (left) learned from GR ChIP-seq data from the IMR90 cell line^47^ and for GR from a SELEX dataset (center). The scatterplot compares the energy coefficients learned from ChIP-seq (*y* axis) and SELEX (*x* axis) data^7^. **b**, Combined specificity (top) and sample-specific TF binding activity (bottom) model learned by jointly analyzing three GR ChIP-seq datasets after treatment with 30 μg kg^−1^, 300 μg kg^−1^ or 3,000 μg kg^−1^ of CORT^51^. The scatterplot (left) compares the energy coefficients as in **a**.

**Extended Data Fig. 9:**
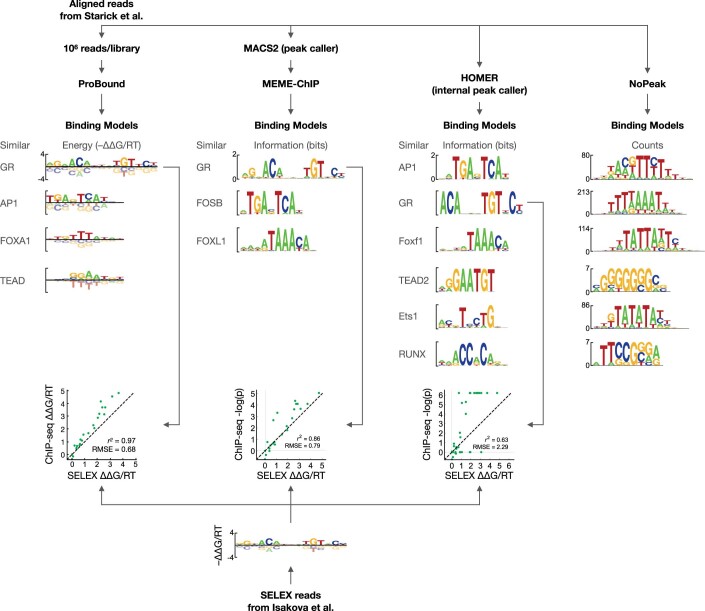
Comparison of GR binding models learned using different algorithms. Top: Binding models inferred by peak-based methods (MEME-ChIP and HOMER) and peak-free methods (ProBound and NoPeak) from the GR ChIP-seq data published in Starick et al. (2015). For MEME-ChIP, the reverse-complement symmetry setting was activated. Bottom: Comparison of ChIP-based and SELEX-based binding models for GR, displayed as in Fig. 5a. Because the binding models generated by MEME-ChIP and HOMER contain base probabilities p, the negative logarithm of these values were compared to the ΔΔG/RT values from the SELEX model. None of the binding models found by NoPeak matched the GR consensus sequence.

To test this approach, we used ProBound to discover the factors driving the selection in glucocorticoid receptor (GR) ChIP-seq data from the IMR90 cell line^47^ (Methods). It found four binding models: one consistent with the GR consensus sequence^48,49^ and three others consistent with known GR co-factors AP-1, FOXA1 and TEAD^47,50^ (Fig. 5a). These models were qualitatively consistent with those discovered using well-established peak-based methods (Extended Data Fig. 9). Inspired by our multi-concentration analysis above, we next set out to quantify the impact that the nuclear concentration of a TF can have on its binding. We did so by jointly analyzing multiple ChIP-seq datasets that probe GR binding in the murine hippocampus after treatment with varying levels of corticosterone (CORT)^51^, an agonist that increases the nuclear concentration of GR (Fig. 5b). The resulting model captured sample-specific activity parameters reflective of GR nuclear concentration that were proportional to CORT concentration (Fig. 5b).

It should be noted that the multi-concentration model was constructed on data where each library was intentionally downsampled to 10^5^ reads or 0.03 reads per kilobase (kb) of genomic sequence on average. Thus, even at extremely low coverage, ChIP-seq data clearly contain sufficient information to reliably infer TF binding models and quantify biologically meaningful cell state parameters. The free-energy parameters of both GR binding models showed good agreement with those from a model trained on in vitro data^7^ (*r*^2^ = 0.97 and *r*^2^ = 0.92, respectively; Fig. 5a,b), suggesting that in vitro and in vivo observations of binding specificity can, in fact, be highly concordant.

## Profiling tyrosine kinase kinetics using Kinase-seq

Biological processes that employ sequence-specific protein–protein interactions are increasingly being studied ﻿with display assays using diverse DNA-templated protein libraries^17,18,52^. Although these methods are profiling such interactions more comprehensively than ever before, interpreting the data remains challenging for many of the same reasons as above. Furthermore, current analytical methods tend to focus on detecting enriched sequence features rather than explicitly estimating binding constants or enzymatic parameters. Given the similarities with SELEX assays, we were motivated to use ProBound to characterize protein sequence recognition.

**Fig. 6:**
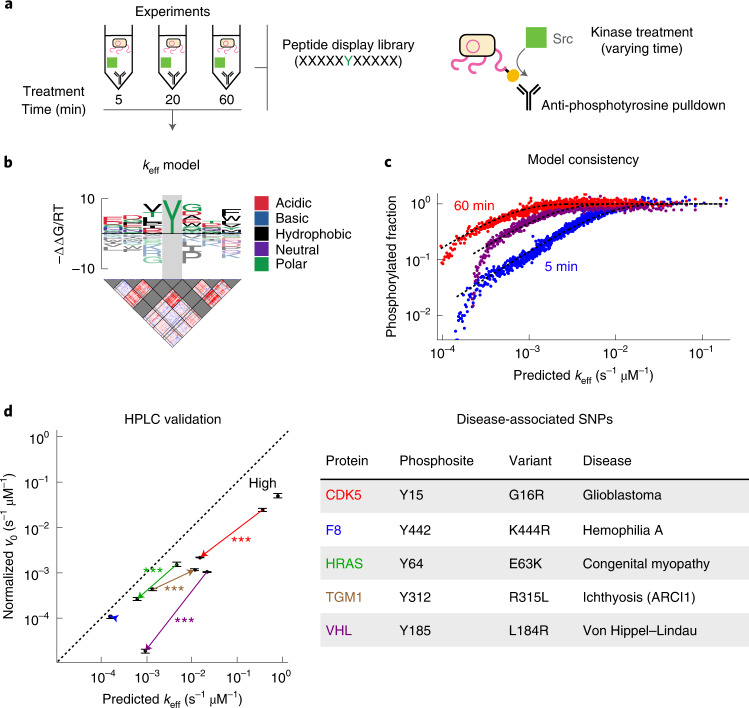
ProBound quantifies sequence-dependent kinetics of the tyrosine kinase c-Src. **a**, Schematic overview of the Kinase-seq assay used to profile the sequence specificity of the tyrosine kinase c-Src. **b**, *k*_eff_ model for c-Src with an energy logo (top) and an interaction matrix (bottom) trained on data from 5 minutes, 20 minutes and 60 minutes of exposure. The central position of the model was fixed to recognize tyrosine (gray). **c**, Comparison of the predicted *k*_eff_ (*x* axis) and phosphorylated fraction (*y* axis) for 5 minutes (blue), 20 minutes (purple) and 60 minutes (red) of exposure to c-Src. Points represent the average observed phosphorylated fraction for 500 probes binned by predicted *k*_eff_. Dashed lines indicate expected value according to the model. **d**, Comparison of the HPLC-measured normalized initial phosphorylation rate *v*_0_ (*y* axis, *n* = 3 technical replicates) and the model-predicted *k*_eff_ (*x* axis) for five disease-associated WT/MUT SNP pairs (arrows) and a peptide predicted to have high activity (Supplementary Table 2). The concentration of c-Src was 500 nM and that of the substrate peptide was 100 μM. Error bars indicate the s.e.m., and *P* values were computed using a two-sided *t*-test (*** indicates *P* < 10^−3^).

**Extended Data Fig. 10:**
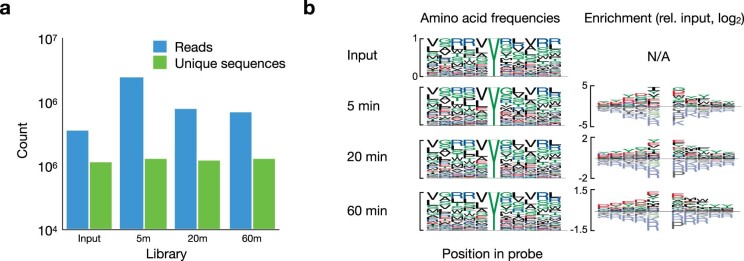
Composition of the Kinase-seq libraries. (a) Bar chart showing the number of reads and unique sequences in each sequencing library. (b) Sequence logos showing the amino acid frequencies (left) and enrichments (right) at each position in each library.

As a proof of concept, we focused on a process critical to many signal transduction pathways in the cell: the phosphorylation of tyrosine residues on proteins. Recently, the substrate sequence preferences of several tyrosine kinases were surveyed with a bacterial display library containing thousands of known kinase substrates^53^. To comprehensively profile the preferences for one of these kinases, c-Src, in an unbiased way, we repeated the assay with a new library design that randomizes ten amino acid residues around a fixed central tyrosine and exposed this library to c-Src for varying durations (Fig. 6a and Methods). After sequencing (Extended Data Fig. 10), we jointly analyzed all time points to learn a model that predicts the sequence-specific catalytic efficiency *k*_eff_, a simple metric that is often used to compare substrates for the same enzyme. Visualizing the inferred efficiency model as a sequence logo (Fig. 6b) revealed a position-specific pattern of favorable residues consistent with the earlier study^53^. The model also accurately captures the observed fraction of phosphorylated peptides over a 100-fold range in *k*_eff_ for all three time points (Fig. 6c).

To validate the model, we used high-performance liquid chromatography (HPLC) to measure the phosphorylation rates for 11 peptides. As genetic variants can impact phosphorylation rates^54^, we used the PTMVars database^55^ to find four disease-associated single-nucleotide polymorphisms (SNPs) that were predicted by our ProBound model to have a large allelic difference. Measurements of their normalized initial phosphorylation rate differed significantly in the direction predicted by the model (Fig. 6d). In addition, there was no measurable difference for a SNP predicted to cause only a small allelic difference for the F8 protein, and a model-defined high-efficiency peptide (Src-high) was indeed the highest. Predictions tracked HPLC measurements over three orders of magnitude in *k*_eff_.

## Discussion

A major goal of this study was to rigorously estimate biophysical parameters from massively parallel sequencing data using machine learning. Although biochemists have measured such parameters for decades, these measurements are generally low-throughput. By contrast, high-throughput sequencing-based analysis tends to focus on the detection of enrichment patterns that only indirectly reflect these quantities. Moreover, modern machine learning methods, such as deep neural networks, tend to yield highly overparametrized black box models whose parameters have no direct biophysical meaning. Here, we showed that, by explicitly modeling the assay process, we can use machine learning to turn DNA sequencers into virtual measurement devices that accurately quantify biophysical parameters. Molecular biologists and computer scientists often address the same question using very different language; for instance, classifier performance and binding free energies are both used to quantify sequence recognition. We hope that approaches such as ours help keep the literature more coherent and inspire direct experimental validation of algorithm performance.

Central to our approach is the observation that some quantities cannot be estimated through pairwise enrichment analysis but only through more structured integration of complementary data. One example is our combinatorial approach to the separation of different TF complexes, which we also extended to methylation-aware binding models. Another is how analyzing the bound, free and input fractions jointly—not pairwise—allows absolute affinities to be measured. Our approach is reminiscent of more traditional biochemical assays, which collect data across different time points, concentrations or fractions and use curve fitting to estimate constants. As we study increasingly complex aspects of sequence recognition—such as the combined impact of sequence, co-factors, DNA methylation and TF concentrations or the integration of in vitro and in vivo perspectives—we foresee that rigorous integration of complementary data along the lines that we have sketched here will become increasingly important. More generally, we anticipate that the accurate and unbiased profiling of sequence recognition that ProBound enables will have many applications in areas of biotechnology where the rational engineering of ligands or substrates is critical.

## Methods

### Overview of the algorithm

For each experiment, the data consist of a count table enumerating the probes in each SELEX round. The core of the algorithm is a statistical model of the experiment that defines the likelihood of a set of model parameters given the count table. On a high level, this likelihood is computed by first defining the probability that each probe is bound in terms of its sequence, then predicting the probe frequencies in each library using a cumulative selection function and, finally, modeling the stochastic sampling of sequencing. The model parameters are estimated from the data through numerical maximization of the likelihood.

### Probabilistic motivation of the binding model

The binding model defines the probability that a probe is bound:

1 $${P}_{{{{\rm{bound}}}}}=\frac{{Z}_{{{{\rm{bound}}}}}}{1+{Z}_{{{{\rm{bound}}}}}}.$$

Here, *Z*_bound_ is the partition function, which can be thought of as a weighted sum over microscopic states. Assuming that, at most, two protein molecules are bound to the probe, the partition function is given by

2 $${Z}_{{{{\rm{bound}}}}}=\mathop{\sum}\limits_{a}\mathop{\sum}\limits_{x}\frac{[{{{{\rm{P}}}}}_{a}]}{{K}_{{{{\rm{D}}}},a}({S}_{x})}+\mathop{\sum}\limits_{a,b}\mathop{\sum}\limits_{x_1,x_2}\frac{[{{{{\rm{P}}}}}_{a}][{{{{\rm{P}}}}}_{b}]}{{K}_{{{{\rm{D}}}},a}({S}_{x_1}){K}_{{{{\rm{D}}}},b}({S}_{x_2})}{\omega }_{a:b}(x_1,x_2),$$

where *a* is a “binding mode” index that denotes protein type; [P_*a*_] is the concentration of protein *a*; *S*_*x*_ is a probe subsequence of length *L*_*a*_ starting at an offset and strand denoted by *x*; *K*_D,*a*_(*S*_*x*_) is the dissociation constant for protein *a* binding *S*_*x*_; and *ω*_*a*:*b*_(*x*_1_, *x*_2_) quantifies the cooperativity between factors *a* and *b* binding at positions *x*_1_ and *x*_2_, respectively. Note that *ω*_*a*:*b*_(*x*_1_, *x*_2_) equals 1 if *a* and *b* bind independently from each other, equals 0 for prohibited conformations and is greater than 1 if the factors bind cooperatively.

It is convenient to express *K*_*D*_ in terms of its value for a references sequence *S*_0_ and a modifying factor quantifying the relative binding strength^27^:

3 $${K}_{{{{\rm{D}}}},a}^{{rel}}({S}_{x})=\frac{{K}_{{{{\rm{D}}}},a}({S}_{x})}{{K}_{{{{\rm{D}}}},a}({S}_{0})}=\exp \left(\frac{{{\Delta }}\Delta {G}_{a}({S}_{x})}{RT}\right).$$

Here, ΔΔ*G*_*a*_(*S*) ≡ Δ*G*(*S*) − Δ*G*(*S*_0_) is the difference in free-energy penalty Δ*G* of binding between *S* and *S*_0_; *R* denotes the ideal gas constant; and *T* is the absolute temperature.

A central goal of our algorithm is to learn how ΔΔ*G*_*a*_(*S*) depends on the sequence. ProBound models this as a sum of additive contributions associated with sequence features *ϕ*:

4 $$-\frac{{{\Delta }}\Delta {G}_{a}({S}_{x})}{RT}=\mathop{\sum}\limits_{\phi \in {{\Phi }}}{\beta }_{a,\phi }{X}_{\phi }({S}_{x})\equiv {{\overrightarrow \beta }}_{a}\cdot {\overrightarrow X}({S}_{x})$$

Here, Φ is the set of sequence features; *β*_*ϕ*_ is the energetic impact of *ϕ*; and *X*_*ϕ*_(*S*_*x*_) is a binary indicator of whether sequence *S*_*x*_ contains *ϕ*. By default, Φ is simply the letter sequence along *S*_*x*_. In this case $\overrightarrow{\beta }$ encodes a position-specific affinity matrix (PSAM)^24,27,56^ with size matching the length of *S*_*x*_. ProBound can also include letter pairs as features, both adjacent (giving dinucleotide interactions for DNA as in, for example, NRLB^25^) and non-adjacent.

Finally, although ProBound is similar to MODER^22^ in that both methods model monomeric and dimeric binding, these methods have several differences: (1) ProBound predicts the quantitative equilibrium binding probability in terms of the biophysically interpretable partition function *Z*_bound_, whereas MODER uses a mixture model and the expectation–maximization algorithm to perform motif discovery; (2) ProBound jontly analyzes all available SELEX rounds, whereas MODER analyzes a single set of bound sequences; (3) MODER allows dimeric interactions to modify the combined position weight matrix for two closely spaced or clashing motifs; and (4) ProBound has broad applicability beyond discovery of dimeric motifs.

### Implementation of binding layer

Although the above derivation provides a motivation for the binding model, it has to be adapted for SELEX experiments. First, it is clear from Eq. (2) that the protein concentration [P_*a*_] and binding constant *K*_D,*a*_(*S*_0_) for a given factor *a* cannot be separately estimated from the data, but only the ratio *α*_*a*_ = [P_*a*_] / *K*_D,*a*_(*S*_0_) can, a quantity that we call the binding mode activity. We similarly define the binding mode interaction activities as *α*_*a*:*b*_ = [P_*a*_][P_*b*_] / *K*_D,*a*_(*S*_0_)*K*_D,*b*_(*S*_0_). Second, because the free protein concentration can vary between SELEX rounds *r*, the activities can take independent values in each round. Third, most experiments are performed in a low-protein-concentration regime where *Z*_bound_ ≪ 1 and *P*_bound_ ∝ *Z*_bound_. Because the data only provide information about the relative rate at which probes are selected, only the relative values of *α*_*a*_ and *α*_*a*:*b*_ are meaningful in this limit. Fourth, although PSAM models can be accurate for close-to-consensus sequences, they severely underestimate the affinity of far-from-consensus sequences, for which non-specific binding is dominant^57^. This can be addressed by including a non-specific binding term *α*_N.S._ in *Z*_bound_. Finally, it is sometimes important to include a factor *ω*_*a*_(*x*) that models biases in binding along the probe. Putting all of this together gives that the partition function in selection round *r* is given by:

5 $$\begin{array}{l}{Z}_{{{{\rm{bound}}}},r}={\alpha }_{{{{\rm{N.S.}}}},r}+\mathop{\sum}\limits_{a}{\alpha }_{a,r}\mathop{\sum }\limits_{x}{\omega }_{a}(x){e}^{{\overrightarrow\beta}_{a}\cdot {\overrightarrow X}({S}_{x})}\\+\mathop{\sum}\limits_{a,b}{\alpha }_{a:b,r}\mathop{\sum }\limits_{x_1,x_2}{e}^{{\overrightarrow\beta }_{a}\cdot {\overrightarrow X}({S}_{x_1})+{\overrightarrow\beta}_{b}\cdot {\overrightarrow X}({S}_{x_2})}{\omega }_{a:b}(x_1,x_2)\end{array}$$

The binding probes typically feature a variable region flanked by constant sequences. The sliding window sum over subsequences *S*_*a*_ can be configured to include *f*_*a*_ letters from the flanking sequences. By default, the sum runs over both strands, but it can be restricted to only one strand (which is useful for modeling RNA and peptides).

### Assay layer

The selection model predicts the relative concentrations *f*_*i*,*r*_ of each binding probe *i* in each selection round *r*. By default, the concentrations in two subsequent rounds are related through an enrichment factor proportional to the binding. It is convenient to express this as

6 $${f}_{i,r}={f}_{i,r-1}{\left({Z}_{{{{\rm{bound}}}},i,r}\right)}^{\rho }{(1+{Z}_{{{{\rm{bound}}}},i,r})}^{\gamma }$$

where *Z*_bound,*i*,*r*_ is the partition function evaluated for probe *i* in round *r*. Experiments conducted in the low-protein-concentration limit are modeled by setting (*ρ*, *γ*) = (1, 0). Binding saturation can be accounted for by setting (*ρ*, *γ*) = (1, −1). Although previous methods have modeled enrichment between a pair of SELEX libraries (such as the linear selection model used by NRLB^25^ and the saturated binding model used by BEESEM to optimally explain the k-mer enrichment in HT-SELEX data^24^), and although the recent DeepSELEX method analyzes multiple SELEX rounds using a multi-layer neural network (although in a way that neither models the thermodynamics of binding nor the cumulative effect of repeated enrichment)^20^, no other method rigorously models how a full SELEX library evolves across multiple selection rounds.

Some experiments (such as *K*_D_-seq; see below) do not use repeated binding enrichment but, rather, derive multiple libraries directly from the input. Such experiments are better modeled using

7 $${f}_{i,r}={f}_{i,0}{\left({Z}_{{{{\rm{bound}}}},i,r}\right)}^{{\rho }_{r}}{(1+{Z}_{{{{\rm{bound}}}},i,r})}^{{\gamma }_{r}}$$

Finally, kinetic experiments that enrich and sequence modified or unmodified probes can be modeled using the constant-rate-enrichment model:

8 $${f}_{i,r}={f}_{i,r-1}\left(\frac{1}{1+{e}^{-\delta }}{e}^{-{Z}_{{{{\rm{bound}}}},i,r}}+\frac{1}{1+{e}^{\delta }}\left(1-{e}^{-{Z}_{{{{\rm{bound}}}},i,r}}\right)\right)$$

Here, *δ*→*∞* and *δ*→−*∞* correspond to the unmodified and modified fractions, respectively.

### Sequencing layer

The sequencing model computes the likelihood of the observed count tables *k*_*i*,*r*_ given the relative concentrations *f*_*i*,*r*_ predicted by the selection model. The counts are assumed to follow a Poisson distribution with expectation value

9 $$E[{k}_{i,r}]={\eta }_{r}{f}_{i,r}$$

Here, the parameter *η*_*r*_ normalizes the relative probe concentration and adjusts to the correct sequencing depth. The (rescaled) likelihood is then

10 $$\log {{{\mathcal{L}}}}=\mathop{\sum}\limits_{r,i}\left[{k}_{i,r}\log ({\eta }_{r}{f}_{i,r})-{\eta }_{r,i}{f}_{i,r}\right]/{k}_{{{{\rm{total}}}}}+{{{\rm{const.}}}}$$

where *k*_total_ is the total number of reads and where the last term is independent of model parameters and can be ignored for the purpose of optimization. Because *f*_*i*,*r*_ is proportional to *f*_*i*,0_, the latter parameter can be optimized analytically and substituted back into Eq. (10), giving

11 $$\log {{{\mathcal{L}}}}=\mathop{\sum}\limits_{r,i}\left({k}_{i,r}\,\log {p}_{r;i}\right)/{k}_{{{{\rm{total}}}}}+{{{\rm{const.}}}}$$

where ${p}_{r;i}={\eta }_{r}{f}_{i,r}/{\sum }_{r^{\prime} }{\eta }_{r^{\prime} }{f}_{i,r^{\prime} }$. Note that Eq. (11) also can be derived by assuming that the counts for each probe follow the multinomial distribution across columns with probability *p*_*r*;*i*_. Also note that, because all unobserved probes have *k*_*i*,*r*_ = 0 and do not contribute to the likelihood, the sum over *i* only runs over the observed probes. This is a major advantage compared to NRLB^25^, where the sum is over all 4^*L*^ probes, with *L* as the number of variable positions. This sum can only be evaluated using dynamic programming, and this restricts NRLB to data from only a single round of affinity-based enrichment in the absence of saturation.

A second advantage of this approach is that it seeks to predict the quantitative count of all observed sequences and give the appropriate weight to both (the relatively rare) high-count sequences and (the much more numerous) low-count sequences. This differs substantially from DeepSELEX^20^ (which builds a multi-library sequence classifier using the top 15,000 sequences and then disregards the sequencing count), DeepBind^19^ (which truncates the sequencing counts of a selected SELEX library into present or absent, generates a synthetic input library and then builds a binary classifier of selected versus input), MODER^22^ (which performs motif discovery within one set of sequences without counts) and BEESEM^24^ (which minimizes the error in the predicted library-wide k-mer frequencies).

Finally, note that Eq. (11) is independent of the initial probe frequencies *f*_*i*,0_, meaning that the initial library need not be random but can consist of genomic DNA fragment or custom-designed sequences.

### Multi-experiment learning

ProBound simultaneously models multiple experiments by computing the likelihood ${{{{\mathcal{L}}}}}_{e}$ of each experiment *e* and then optimizing the combined likelihood

12 $$\begin{array}{rcl}\log {{{\mathcal{L}}}}&=&{\sum }_{e}\log {{{{\mathcal{L}}}}}_{e}\end{array}$$

The precise way in which the likelihood ${{{{\mathcal{L}}}}}_{e}$ is evaluated can be tailored to the details of each experimental design:

1. A different configuration of binding modes and their interactions can be chosen for each experiment when computing *Z*_bound_ when desired.
2. The binding mode (and interaction) activities can either take independent values *α*_*a*,*e*_ in each experiment or be constrained to ${\alpha }_{a,e}={[{{{{\rm{P}}}}}_{a}]}_{e}{\alpha }_{a}$, where *α*_*a*_ is the global activity of binding mode *a* and [P_*a*_] is a set parameter. The latter is useful when integrating experiments conducted at different protein concentrations or in kinetic assays where [P_*a*_] is set to the treatment time.
3. Chemical modifications are encoded by expanding the alphabet and transliterating letters to appropriate experiments. For example, meCpG modifications can be encoded using the alphabet ACcGgT and the complementarity rules A ↔ T, C ↔ G and c ↔ g, expanding the feature set Φ of the binding model to include the additional letters and performing the transliteration CG → cg for methylated probes.

To our knowledge, no other methods have similar functionality for jointly analyzing multiple complementary SELEX datasets.

### Regularization

Three regularization terms were included to avoid overfitting and to improve the stability of the numerical optimization. The first was a *L*_2_ regularization term for the parameter vector

13 $$\begin{array}{r}\overrightarrow{\theta }=\{{\beta }_{\phi },\log {\alpha }_{a},\log {\alpha }_{a:b},\log {\omega }_{a}(x),\log {\omega }_{a:b}(x_1,x_2),\log {\eta }_{r}\}\end{array}$$

with weight *λ*. The second term was inspired by the Dirichlet distribution, which commonly is used as a prior for probability parameters. Thus, for each feature *ϕ*, we identified all features Φ^*c*^(*ϕ*) that are of the same class *c* (monomer, or dimer with the same spacing) and located at the same position within the binding site, and then we defined a feature probability

14 $$\begin{array}{rcl}p(\phi) &=& {e}^{{\beta }_{\phi }} \left({\mathop{\sum}\limits_{\phi ^{\prime} \in {{{\Phi }}}^{c}(\phi )}{e}^{\beta_{\phi^\prime}}} \right)^{-1}\end{array}$$

The regularization term is then computed as the rescaled log-PDF of *p*(*ϕ*) in the Dirichlet distribution

15 $$\frac{{k}_{{{{\rm{Dirichlet}}}}}}{{k}_{{{{\rm{total}}}}}}\mathop{\sum}\limits_{\phi }\log p(\phi )$$

where *k*_Dirichlet_ is analogous to a pseudocount. The final regularization term in the likelihood is defined as

16 $$\mathop{\sum}\limits_{i}\left({e}^{{\theta }_{i}-{\theta }_{\max }}+{e}^{-{\theta }_{i}-{\theta }_{\max }}\right)$$

and introduces an exponential barrier (by default ${\theta }_{\max }=40$) that prevents the optimizer from failing or getting trapped in regions with large numerical errors.

### Procedure for setting *k*_Dirichlet_

The importance of the Dirichlet regularizer in Eq. (15) is set by *k*_Dirichlet_. For fits with all-by-all interactions, the inferred coefficients tended to be unstable for small values of *k*_Dirichlet_. Although increasing *k*_Dirichlet_ stabilizes the coefficients, they shrink toward 0 when *k*_Dirichlet_ is excessively large. We, thus, developed a procedure for setting *k*_Dirichlet_ and applied it uniformly in all analyses that included dinucleotide or all-by-all interactions. In this procedure, we ran ProBound using a wide range of Dirichlet weights (*k*_Dirichlet_ ∈ {0, 10, 20, 50, 100, 200, 500, 1,000, 2,000}), fixed the monomer coefficients ${\overrightarrow{\beta }}_{{{{\rm{mono}}}}}$ and dimer coefficients ${{\overrightarrow \beta }}_{{{{\rm{di}}}}}$ in each resulting model using the mismatch gauge (see below) and computed the pairwise Pearson correlation *r*^2^ between the inferred ${{\overrightarrow\beta }}_{{{{\rm{di}}}}}$ for different values of *k*_Dirichlet_. The resulting matrix *r*^2^(*k*_1_, *k*_2_), where *k*_1_ and *k*_2_ are values of *k*_Dirichlet_, had a block-like structure where ${{\overrightarrow \beta }}_{{{{\rm{di}}}}}$ was highly correlated for large values of *k*_1_ and *k*_2_ but only weakly correlated when *k*_1_ or *k*_2_ was small. We considered the coefficients to have stabilized when *r*^2^ > 0.8 between a model and the model with the next-smaller value of *k*_Dirichlet_. Using this procedure, we fixed *k*_Dirichlet_ to be 0 for the Hth-Exd-Ubx analysis (Fig. 3b), 0 for the ATF4/CEBP*γ* EpiSELEX-seq analysis (Fig. 3c), 0 for the CEBP*γ*:CEBP*γ* multi-EpiSELEX-seq analysis (Fig. 3e), 200 for the RBFOX2 analysis (Extended Data Fig. 7f), 200 for the single-experiment Dll analyses (Fig. 4b), 1,000 for the multi-experiment Dll analyses (Extended Data Fig. 7c–e) and 50 for the Src analysis (Fig. 6b). *k*_Dirichlet_ was set to 20 in all analyses that lacked interactions—namely, the SELEX benchmarking (Fig. 2), the CAP-SELEX analyses (Extended Data Figs. 2c and 3) and the ChIP-seq analysis (Fig. 5).

### Model optimization scheme

To estimate the model parameters, ProBound uses the quasi-Newton optimization method L-BFGS to minimize the loss function. As gradient-based methods cannot guarantee convergence to the global minimum, we developed a heuristic method that escapes common local minima. Specifically, given an optimal binding model, closely related but suboptimal models can be generated by (1) shifting the motif to the left or right, (2) extending or shrinking the motif to the left or right and (3) increasing or deceasing the flank length^25^. Thus, given that L-BFGS converges at a minimum, our method explores the above transformations to find the model with the optimal footprint.

More precisely, ProBound optimizes the loss function by first restricting it to include only the first binding mode (and non-specific binding) and optimizing this model and then sequentially including and optimizing additional binding modes (and interactions as they become possible). As each new binding mode *a* (or interaction *a*:*b*) is included and optimized, the algorithm takes seven substeps: (1) heuristic adjustment of *α*_*a*_ (or *α*_*a*:*b*_) so that it is expected to contribute to 5% to *Z*_bound_; (2) freezing the values of all model parameters; (3) unfreezing and optimizing *η* to avoid shocks from incorrectly predicted sequencing depth; (4) unfreezing and optimizing the monomer features in ${{\overrightarrow \beta }}_{a}$ mode to give an initial binding model (*ω*_*a*:*b*_ (x_1_,x_2_)﻿ is unfrozen and optimized for interactions); (5) greedy exploration of alternative binding models with different frame shift (shifting the recognized sequence features to left or right), footprint (expanding the region of feature recognition to the left and/or right) or flank length (including subsequences located further into the fixed flanking regions when computing *Z*_bound_); (6) sequential unfreezing and optimization of dimer features and *ω*_*a*_(*x*) if applicable; and (7) unfreezing of all model parameters. At each substep, L-BFGS is used to optimize the unfrozen parameters. By default, the parameters are seeded with small random numbers, but the binding modes can also optionally be seeded using International Union of Pure and Applied Chemistry (IUPAC) codes. Additional constraints can be imposed on the parameters to implement reverse-complement symmetric binding modes or translationally symmetric interactions.

### Gauge fixing

Models with pairwise letter interactions are over-parametrized, meaning that an infinite set of parameter values ${\overrightarrow \beta }$ encode the same sequence specificity. Specifically, for any binding site sequence *S*, ${\overrightarrow \beta }\cdot {\overrightarrow X}(S)$ is invariant under transformations of the form

17 $${\beta }_{\phi }\to {\beta }_{\phi }+A\quad \forall \phi \in {{{\Phi }}}_{{{{\rm{mono}}}}}({x}_{1})$$

18 $${\beta }_{\phi }\to {\beta }_{\phi }-A\quad \forall \phi \in {{{\Phi }}}_{{{{\rm{di}}}}}({x}_{1},{x}_{2},n)$$

where Φ_mono_(*x*_1_) is the set of monomer features at position *x*_1_; Φ_di_(*x*_1_, *x*_2_, *n*) is the set of dimer features connecting positions *x*_1_ and *x*_2_ and with *n* at *x*_2_; and *A* is the transformation coordinate. For visualization and model comparison purposes, it is convenient to select one representative model for each sequence specificity (analogous to gauge fixing in physics). Here, we use a convention that we call the ‘mismatch gauge’. In this convention, the coefficients are such that, first, only one monomer coefficient contributes for single-edit variations of reference sequence *S*_0_, and, second, at most one of the dimer coefficients contributes for each double-edit variation of *S*_0_. After imposing mutation gauge, the resulting PSAMs were visualized using standard energy logos^27^, and the interaction coefficients were displayed using heat maps.

### Benchmarking ProBound

#### Model training

To benchmark ProBound, we first curated a training database of published TF SELEX datasets^7,8,10,12,13,28–30^. Although this database contained 2,272 datasets, Yang et al.^30^ contained re-sequenced libraries from Jolma et al.^28^, and, thus, the database contained 1,767 unique experiments. Datasets with low sequencing depth or low enrichment were filtered out as described below, giving 2,116 datasets (1,632 experiments).

We next developed a uniform computational pipeline to analyze each dataset. This was complicated by experimental differences between the SELEX platforms, including the number of selection rounds, selection strength and sequencing depth. Furthermore, several artifacts are known to impact HT-SELEX datasets, including contamination between wells, inconsistent selection between rounds and sequence biases^6,19,23,25,28^. Although such challenges can be overcome using manual inspection^19,28^, we instead chose to develop a fully automated system. This system first uses ProBound to analyze each dataset (subsampled to 100,000 reads per sequencing library) using three different settings (that differ in the number of binding modes and in how non-specific binding is modeled; see Extended Data Methods) and then prunes each fit to retain only the most relevant binding mode and, finally, selects the setting that produced the best-performing binding model (based only on the training data).

#### Model pruning

For each fit generated by ProBound, one binding mode typically captured the TF sequence specificity, and the other typically had small values or encoded platform-specific artifacts, such as sequence bias or contamination. Although identifying the biophysically relevant binding mode manually is straightforward in most cases, we wanted to automate this process and, therefore, developed a quality score that ranks and selects the most relevant binding mode:

19 $${r}_{{{{\rm{mode}}}}}^{2}+\log {I}_{{{{\rm{mono}}}}}$$

Here, ${r}_{{{{\rm{mode}}}}}^{2}$ is the the Pearson correlation (across the SELEX probes in the training dataset) of the log-transformed binding affinity predicted by the mode (plus an optimized non-specific term) and the log-transformed binding predicted by the full fit, and *I*_mono_ is the information content of the mononucleotide coefficients after imposing the mismatch gauge. This score favors the binding mode that contributes the most to the final prediction and has the highest specificity. Conversely, it disfavors binding modes corresponding to sequence bias (which can affect many probes but typically have low information content) and contamination (which typically impacts few probes but can give rise to highly specific binding modes). We, thus, selected the binding mode with the highest quality score for downstream analysis.

#### Model selection

We next compared the binding models learned using the three settings. Although very similar in most cases, poor models were occasionally observed having suboptimal motif shifts or encoding the aforementioned artifacts. To automatically select the best model, we developed the quality score *S*_training_, which measures model performance in predicting the training data. As the heterogeneity of the training data made it difficult to quantify this performance using a single measure, *S*_training_ was defined to be the average of six sub-scores that quantify different aspects of model performance:

20 $$\begin{array}{rcl}{S}_{{{{\rm{training}}}}}=&&{{{\rm{mean}}}}\left(\left\{{F}_{{{{\rm{logit}}}}}({r}_{{{{\rm{fit,8mer}}}}}^{2};0.5),{F}_{{{{\rm{logit}}}}}({R}_{{{{\rm{fit,affinity}}}}}^{2},0.95),{F}_{\log }({f}_{{{{\rm{fit,affinity}}}}};5.0),\right.\right.\\&& {F}_{{{{\rm{logit}}}}}({R}_{{{{\rm{scoring,training}}}}}^{2};0.95),\,{F}_{\log }(MAF{R}_{{{{\rm{scoring,training}}}}};5.0),\\&& \left.\left.{F}_{\log }({I}_{{{{\rm{scoring,mono}}}}};3.0)\right\}\right)\end{array}$$

where the functions ${F}_{{{{\rm{logit}}}}}(x;{x}_{0})={{{\rm{expit}}}}\left({{{\rm{logit}}}}(x)-{{{\rm{logit}}}}({x}_{0})\right)$ and ${F}_{\log }(x;{x}_{0})={{{\rm{expit}}}}\left(\log (x)-\log ({x}_{0})\right)$ map the metric *x* to the unit interval such that the threshold *x*_0_ maps to 0.5. Here,

- ${r}_{{{{\rm{fit,8Mer}}}}}^{2}$ was computed by first using the full ProBound model to predict the training count table, then counting the number of occurrences ${n}_{{{{\rm{8mer}}}}}^{{{{\rm{obs/pred}}}}}(k,r)$ of each 8mer *k* in each round *r* of the of the observed and predicted count tables and then computing the observed and predicted 8mer enrichment between the first and last round using
  21 $${f}_{{{{\rm{8mer}}}}}^{{{{\,\rm{obs/pred}}}}}(k)=\frac{1}{{r}_{{{{\rm{last}}}}}-{r}_{{{{\rm{first}}}}}}\log \left(\frac{1+{n}_{{{{\rm{8mer}}}}}^{{{{\rm{obs/pred}}}}}(k,{r}_{{{{\rm{last}}}}})}{1+{n}_{{{{\rm{8mer}}}}}^{{{{\rm{obs/pred}}}}}(k,{r}_{{{{\rm{first}}}}})}\right)$$
  and, finally, computing the Pearson correlation between ${f}_{{{{\rm{8mer}}}}}^{{{{\,\rm{obs}}}}}$ and ${f}_{{{{\rm{8mer}}}}}^{{{{\,\rm{pred}}}}}$.
- ${R}_{{{{\rm{fit,affinity}}}}}^{2}$ and *f*_fit,affinity_ were computed by first using the full ProBound model to predict the training count table. Then, for each pair of subsequent rounds *r* and next(*r*) (ignoring rounds with fewer than 10,000 reads), the probes were sorted (conjointly in the observed and predicted tables) by the predicted enrichment between the rounds. The probes were then divided into bins *i* associated with the observed and predicted probe counts ${n}_{{{{\rm{bin}}}}}^{{{{\rm{obs/pred}}}}}(i,r)$ such that ${n}_{{{{\rm{bin}}}}}^{{{{\rm{obs}}}}}(r)+{n}_{{{{\rm{bin}}}}}^{{{{\rm{obs}}}}}({{{\rm{next}}}}(r))=1000$ in each bin. After computing the observed and predicted enrichment using
  22 $${f}_{{{{\rm{bin}}}}}^{{{{\,\rm{obs/pred}}}}}(i;r)=\frac{1}{{{{\rm{next}}}}(r)-r}\log \left(\frac{1+{n}_{{{{\rm{bin}}}}}^{{{{\rm{obs/pred}}}}}(i,{{{\rm{next}}}}(r))}{1+{n}_{{{{\rm{bin}}}}}^{{{{\rm{obs/pred}}}}}(i,r)}\right)$$
  we finally computed the metrics
  23 $${R}_{{{{\rm{fit,affinity}}}}}^{2}={R}_{k}^{2}\,{\max }_{r}\left({f}_{{{{\rm{bin}}}}}^{{{{\,\rm{obs}}}}}(i;r),{f}_{{{{\rm{bin}}}}}^{{{{\,\rm{pred}}}}}(i;r)\right)$$
  24 $${f}_{{{{\rm{fit,affinity}}}}}={\max }_{r}\left(\frac{{\max }_{i}{f}_{{{{\rm{bin}}}}}^{{{{\,\rm{obs}}}}}(i;r)}{{\min }_{i}{f}_{{{{\rm{bin}}}}}^{{{{\,\rm{obs}}}}}(i;r)}\right)$$
  where ${R}_{i}^{2}$ denotes the coefficient of variation evaluated across bins *i*.
- ${R}_{{{{\rm{scoring,training}}}}}^{2}$ and *MAFR*_scoring,training_ were computed using the same method that was used to quantify generalization performance in predicting testing SELEX data (see below) but, instead, predicting the training data.
- *I*_scoring,mono_ is the information content of the scoring model, computed using the monomer coefficients after imposing the mismatch gauge.

Finally, as each of the re-sequenced experiments had two associated fits (based on data from Jolma et al.^28^ and Yang et al.^30^, respectively), we selected the fit with the best training performance *S*_training_ for benchmarking purposes.

### Evaluation of model performance

To benchmark the resulting binding models, we curated a testing database of published SELEX (same as training database), PBM^58–60^ and ENCODE ChIP-seq^32^ datasets. We then quantified the ability of the above binding models to predict the testing data. Binding models and testing data were matched by TF and species; if no match was found, the matching criteria were expanded to consider orthologous human and mouse TFs. For comparison, we also downloaded binding models from the JASPAR, DeepBind and HOCOMOCO databases, the original HT-SELEX TF binding survey and from the recently published DeepSELEX method^19,20,28,33,34^, and we repeated all analysis using these models. For the SELEX dataset predictions, comparisons were skipped if either the ProBound model or the downloaded model were known to be trained on the testing dataset in question (or other datasets from the same laboratory).

For the SELEX and PBM experiments, we used the binding models to predict the total affinity (denoted *x*_*i*_) for each probe *i* and quantified how well these predictions agree with the measured binding *y*_*i*_. For the SELEX experiments, the signal consisted of the probe count enrichment *k*_*i*,*r*+1_ / *k*_*i*,*r*_ between subsequent SELEX rounds (with maximum normalized to 1). For the PBM experiments, the background-subtracted and minimum–maximum normalized binding signal was used. For both platforms, we encountered two challenges. First, the measurements for individual probes were too noisy to quantify model performance accuracy (for SELEX, typical sequences were observed just once; for PBM, the signal depends strongly on the position of the binding site in the probe, which varies). Inspired by earlier PBM analyses that removed position bias by considering the 8mer-binned median signal^31,56^, we sorted and binned the probes using *x*_*i*_ (with bin size 500 for SELEX and 10 for PBM) and then computed the binned signal *y*_*i*_ (using the bin-averaged enrichment, with pseudocount 1, for SELEX, and the median signal for PBM). Second, binding signals can be distorted by experimental artifacts, such as binding saturation, background and non-specific binding not modeled by the model. To correct for such distortions, *x*_*i*_ was transformed using the binding saturation function:

25 $${\hat{y}}_{i}=\frac{{\beta }_{0}}{1+{({\beta }_{{{{\rm{C}}}}}({x}_{i}+{\beta }_{{{{\rm{NSB}}}}}))}^{-1}}$$

Here, *β*_0_ sets the scale, *β*_C_ > 0 sets the concentration and *β*_NSB_ sets the non-specific binding. These parameters were estimated by minimizing ${\sum }_{i}{[\log ({y}_{i}/{\hat{y}}_{i})]}^{2}$ for SELEX (with *β*_0_ > 0 and *β*_NSB_ > 0) and ${\sum }_{i}{({y}_{i}-{\hat{y}}_{i})}^{2}$ for PBM (for which *y*_*i*_ can be negative). Model quality was then quantified using the coefficient of determination *R*^2^ of *y*_*i*_ and ${\hat{y}}_{i}$ (on a logarithmic scale for SELEX) and the MAFR, which is defined as $(\mathop{\max }\limits_{i}\,{y}_{i})/{y}_{{{{\rm{bg}}}}}$ where *y*_bg_ is the weakest signal detected by the model. To estimate *y*_bg_, we first defined a set of (binned) probes predicted to be bound as ${\hat{y}}_{i} > 1.25\,{Q}_{1}(\hat{y})$ (where *Q*_1_ is the first quartile) and then defined *y*_bg_ to be the smallest value of *y*_*i*_ identifying the bound set at 5% false discovery rate (FDR). For multi-round SELEX experiments, *R*^2^ and the effective range were computed for all rounds, and the largest values were recorded.

For the ChIP-seq experiments, we quantified model performance using the AUPRC in classifying binding peak versus background sequences. To get the peak sequences, we downloaded narrowPeak files from the ENCODE portal (see below) and extracted the genome sequence from the 500 peaks with the strongest enrichment. To generate the background set, we shifted the peak interval one peak length to the left and right and extracted the genome sequences.

### Filtering of SELEX training datasets

We first curated a database of published SELEX experiments and downloaded the associated raw sequencing data^7,8,10,12,13,28–30^. Methylated SELEX experiments were not considered. For each experiment, we downsampled the sequencing libraries to contain, at most, 100,000 reads and tabulated the probe counts in each SELEX round. We then filtered out low-quality experiments using three criteria. First, low-coverage experiments were removed by requiring at least two rounds to have at least 10,000 reads. Second, experiments were discarded if no sequencing library before round three had 10,000 or more reads. Third, experiments with low enrichment were discarded. The enrichment was quantified by first tabulating the frequencies *p*(*k*, *r*) (using pseudocount 5) of all 5mers *k* in each SELEX round *r* and then, for each pair of rounds *r*_*i*_ and *r*_*j*_ with 10,000 or more reads, computing the rescaled Kullback–Leibler (KL) divergence

26 $$\begin{array}{rcl}{D}_{{{{\rm{KL}}}}}({r}_{2},{r}_{1})&=&\frac{1}{{r}_{2}-{r}_{1}}\mathop{\sum }\limits_{k}p(k,{r}_{2})\,{\log }_{2}\,\frac{p(k,{r}_{2})}{p(k,{r}_{1})}\end{array}$$

Only experiments with rescaled KL divergence exceeding 0.01 for at least one combination of rounds were retained.

### Scoring of binding probes

In quantifying generalization performance, we predicted the occupancy of DNA sequences using both the ProBound binding models and previously published models. For DeepBind, we exponentiated the scores returned from the deepbind scoring tool, which is proportional to binding affinity. For JASPAR and original HT-SELEX TF survey, the binding models were position–frequency matrices (containing counts). These were first converted to position probability matrices (PPMs, using a pseudocount of 1), which were then used to compute the binding probability at each offset in the sequence. The occupancy was then defined to be the sum of the binding probabilities. For HOCOMOCO, the binding models were PPMs, and the occupancies were computed as described above. For DeepSELEX, which outputs the difficult-to-interpret quantity $A=\max (\overrightarrow{p}({R}_{4}))+\max (\overrightarrow{p}({R}_{3}))-\max (\overrightarrow{p}({R}_{0}))\in [-1,2]$ (where $\overrightarrow{p}({R}_{k})$ is a vector containing the predicted probability for SELEX round *k* along the scored sequence), the values were transformed using the linear map (*A* + 1) / 3 to occupy [0, 1].

### ENCODE ChIP-seq datasets

ENCODE datasets were downloaded in December 2018 using this query string.

### Binding by multi-protein complexes

#### ProBound analysis

ProBound was configured to jointly analyze SELEX experiments performed with different combinations of TFs, as described in the Extended Data Methods. In the case of Hth-Exd-Ubx, we analyzed published SELEX-seq data for Exd-Ubx, Hth, Exd and Ubx. In addition, we performed a SELEX-seq assay for Hth-Exd-Ubx (see below). CAP-SELEX data for human TF pairs were analyzed jointly with matched single-TF HT-SELEX data as described in the Extended Data Methods and Supplementary Table 3.

#### Experimental protocol

The Hth-Exd-Ubx SELEX experiment was carried out following previously published methods^8,61^. In brief, after expressing and purifying the wild-type homeodomain proteins, a final concentration of 50 nM was assembled, incubated with excess DNA (10–20 fold) for 30 minutes and loaded onto an EMSA gel. A DNA library with 30 randomized bases was used. The TF-bound fraction was isolated from the gel and amplified and either subjected to another round of enrichment or prepared for sequencing. Three rounds of enrichment were performed. After each selection round, the DNA was extracted from the gel and amplified by using Ilumina’s small RNA primer sets. Sequencing barcodes were added in a five-cycle PCR step, and the final library was gel-purified using a native TBE gel before sequencing. Libraries were sequenced at the New York Genome Center using separate lanes on an Illumina HiSeq 2000 sequencing machine.

### Effect of DNA methylation

#### ProBound analysis

ProBound learns methylation-aware binding models by jointly analyzing normal and methylated SELEX libraries after encoding the methylation state of each base pair using an extended alphabet (Extended Data Fig. 4a and configuration in Extended Data Methods). Encoding methylation status in this manner allows us to infer the position-specific free-energy impact of such chemical modifications. For the ATF4/CEBP*γ* homodimers and heterodimers, we jointly analyzed two published EpiSELEX-seq experiments for ATF4 and CEBP*γ* and a new EpiSELEX-seq experiment that included both ATF4 and CEBP*γ*. We also generated EpiSELEX-seq data for CEBP*γ* in combination with the chemical modifications meCpG, 5hmC and 6mA.

#### Experimental protocol

ATF4 protein purification and EpiSELEX-seq experiments were performed as described previously^13^. Purified CEBP*γ* protein was kindly donated by the Lomvardas laboratory at the Zuckerman Institute at Columbia University. To generate randomized 5hmC or 6mA libraries, single-stranded oligos with a 16-bp randomized region were ordered from TriLink Biotechnologies, substituting (1) deoxycytidine triphosphate (dCTP) with deoxy-(5hm)-cytidine triphosphate (d5hmCTP) or (2) deoxyadenosine triphosphate (dATP) with deoxy-(6m)-adenosine triphosphate (d6ATP) during the synthesis step. For double-stranding, a standard mix of deoxy-nucleotides was used, resulting in hemi-modified libraries. meCpG libraries were generated by enzymatic treatment with M.SssI (NEB) as described previously^13^. The library sequences consisted of left and right constant adapters (GGTAGTGGAGG- and -CCAGGGAGGTGGAGTAGG, respectively) flanking a library specific barcode and a 16-bp randomized sequence:

- no modification: -TGGG-CCTGG-N16-
- meCpG: -GCAC-CCTGG-N16-
- 5hmC-Library: -CAGT-CCTGG-N16- (5hmC instead of C in 16N)
- 6mA-Library: -AGTG-CCTGG-N16- (6mA instead of A in 16N)

#### GLM analysis of ATF4 and CEBP*γ* ChIP data

To estimate the effect of DNA methylation on in vivo AFT4 and CEBP*γ* binding, we first scanned the genome for close-to-consensus motif matches *i* with CG at positions predicted by the model to have strong methylation readout: TGACGTCA and TGACGTCG for ATF4:AFT4; TTGCGCAA for CEBP*γ*:CEBP*γ*; and TTGCGTCA and TTGCATCG for CEBP*γ*:ATF4. We next downloaded aligned ATF4 and CEBP*γ* ChIP-seq reads and matched input from ENCODE (ENCFF872NFM, ENCFF801LQC and ENCFF713PVH), extended the alignments to 125 bp and computed the genome coverages (*k*_ATF4,*i*_, *k*_CEBP*γ*,*i*_, *k*_Input,*i*_) at each motif match. The DNase-seq coverage (*k*_DNase,*i*_, ENCFF971AHO) and bisulfite sequencing methylation status (*f*_meCpG,*i*_, ENCSR765JPC, binarized using 20% and 80% thresholds and keeping matches with at least ten reads) were also recorded. We finally modeled the ATF4 and CEBP*γ* ChIP-seq coverage at the relevant motif matches (excluding CEBP*γ*:CEBP*γ* matches for ATF4 and ATF4:ATF4 matches for CEBP*γ*) using two separate binomial generalized linear models:

27 $${k}_{{{{\rm{ChIP,i}}}}} \sim {{{\rm{Binomial}}}}\left({k}_{{{{\rm{ChIP}}}},i}+{k}_{{{{\rm{Input}}}},i},\frac{{e}^{{\eta }_{i}}}{1+{e}^{{\eta }_{i}}}\right)$$

28 $${\eta }_{i}={\beta }_{0,a}+{k}_{{{{\rm{DNase}}}},i}\,{\beta }_{{{{\rm{DNase}}}}}+{f}_{{{{\rm{meCpG}}}},i}\,{\beta }_{{{{\rm{meCpG}}}},a}$$

In this model, *β*_0,*a*_ encodes the relative affinity of motif *a*; *β*_DNase_ encodes the impact of DNA accessibility; and *β*_meCpG_ encodes the impact of DNA methylation for motif *a* and is the sought-after variable. The significance of the methylation readout was assessed using an *F*-test (Supplementary Table 4). For TGACGTCG, we assumed that the methylation readout of the two CGs contribute independently and that the readout of the central CG can be estimated using the sequence TGACGTCA.

### Inferring absolute *K*_D_s

The *K*_D_-seq assay incubates a TF (or other protein) with a library of DNA probes (or RNA or peptide probes), separates the bound and free probes and sequences the input (I), bound (B) and free (F) fractions. In equilibrium, the probability that probe *i* is bound or free is given by

29 $$\begin{array}{rcl}p({{{\rm{B}}}}| i)&=&\frac{{[{{{{\rm{DNA}}}}}_{i}]}_{{{{\rm{B}}}}}}{{[{{{{\rm{DNA}}}}}_{i}]}_{{{{\rm{I}}}}}}=\frac{{[{{{\rm{P}}}}]}_{{{{\rm{F}}}}}}{{[{{{\rm{P}}}}]}_{{{{\rm{F}}}}}+{K}_{{{{{\rm{D}}}}}_{i}}}\\ p({{{\rm{F}}}}| i)&=&\frac{{[{{{{\rm{DNA}}}}}_{i}]}_{{{{\rm{F}}}}}}{{[{{{{\rm{DNA}}}}}_{i}]}_{{{{\rm{I}}}}}}=\frac{{K}_{{{{\rm{D}}}},i}}{{[{{{\rm{P}}}}]}_{{{{\rm{F}}}}}+{K}_{{{{\rm{D}}}},i}}\end{array}$$

where ${[{{{{\rm{DNA}}}}}_{i}]}_{{{{\rm{I}}}}}$, ${[{{{{\rm{DNA}}}}}_{i}]}_{{{{\rm{B}}}}}$ and ${[{{{{\rm{DNA}}}}}_{i}]}_{{{{\rm{F}}}}}$ are the probe concentrations in the input, free and bound libraries; [P]_F_ is the free protein concentration; and *K*_D,*i*_ is the dissociation constant that we want to measure. The sequencer does not measure *p*(B∣*i*) or *p*(F∣*i*) directly but, rather, gives the probe counts *k*_*i*,I_, *k*_*i*,B_ and *k*_*i*,F_. The expectation values of these counts are given by

30 $$\begin{array}{rcl}\frac{E[{k}_{i,{{{\rm{I}}}}}]}{{k}_{{{{\rm{I}}}}}}&=&\frac{{[{{{{\rm{DNA}}}}}_{i}]}_{{{{\rm{I}}}}}}{{[{{{\rm{DNA}}}}]}_{{{{\rm{I}}}}}}=p(i)\\ \frac{E[{k}_{i,{{{\rm{B}}}}}]}{{k}_{{{{\rm{B}}}}}}&=&\frac{{[{{{{\rm{DNA}}}}}_{i}]}_{{{{\rm{B}}}}}}{{[{{{\rm{DNA}}}}]}_{{{{\rm{B}}}}}}=p(i| {{{\rm{B}}}})\\ \frac{E[{k}_{i,{{{\rm{F}}}}}]}{{k}_{{{{\rm{F}}}}}}&=&\frac{{[{{{{\rm{DNA}}}}}_{i}]}_{{{{\rm{F}}}}}}{{[{{{\rm{DNA}}}}]}_{{{{\rm{F}}}}}}=p(i| {{{\rm{F}}}})\end{array}$$

where [DNA]_I_, [DNA]_B_ and [DNA]_F_ are the DNA concentrations in the respective fractions and *k*_I_, *k*_B_ and *k*_F_ are the sequencing depths of the libraries, which are treated as fixed experimental setting. To estimate the dissociation constants, note that

31 $$\frac{{K}_{{{{\rm{D}}}},i}}{{[{{{\rm{P}}}}]}_{{{{\rm{F}}}}}}=\frac{p(F| i)}{p(B| i)}=\frac{p(i| F)p(F)}{p(i| B)p(B)}$$

where *p*(*B*) and *p*(*F*) are the net fractions of DNA that is bound and free. Intuitively, these can fractions can be estimated from the data by finding the values that make the observed probabilities in Eq. (30) satisfy the sum rule:

32 $$p(i)=p(i,F)+p(i,B)=p(i| F)p(F)+p(i| B)p(B)$$

ProBound can be configured to learn a *K*_*D*_ model by analyzing the probe frequencies in the input, bound and free libraries (*r* = {I, B, F}). Specifically, configuring ProBound to use the non-cumulative enrichment model (Eq. (7)) with *ρ*_*r*_ = {0, 1, 0} and *γ*_*r*_ = {0, − 1, − 1} and restricting the activities to be constant across columns implements the binding probabilities in Eq. (29). With these settings, the dissociation constant is

33 $${K}_{{{{\rm{D}}}},i}={[{{{\rm{P}}}}]}_{{{{\rm{F}}}}}/{Z}_{{{{\rm{bound}}}},i}$$

Here, the free-protein concentration can be computed using

34 $${[{{{\rm{P}}}}]}_{{{{\rm{F}}}}}={[{{{\rm{P}}}}]}_{{{{\rm{T}}}}}-{[{{{\rm{DNA}}}}]}_{{{{\rm{I}}}}}\,p({{{\rm{B}}}})$$

where [P]_T_ is the total protein concentration. In most cases, [P]_F_ is close to the more readily measured [P]_T_ due to the low average affinity of randomized ligand libraries. However, here, *p*(B) is implicitly estimated by ProBound and can be computed by equating the expected counts in ProBound

35 $$E[{k}_{i,{{{\rm{I}}}}}]={\eta }_{I}\,{f}_{i,{{{\rm{I}}}}}$$

36 $$E[{k}_{i,{{{\rm{B}}}}}]={\eta }_{B}\,{f}_{i,{{{\rm{I}}}}}\,p({{{\rm{B}}}}| i)$$

37 $$E[{k}_{i,{{{\rm{F}}}}}]={\eta }_{F}\,{f}_{i,{{{\rm{I}}}}}\,p({{{\rm{F}}}}| i)$$

with the corresponding expectation values in Eq. (30), computing the bound-to-input ratio, and using Bayes’ theorem to simplify, giving

38 $$p({{{\rm{B}}}})=\frac{{k}_{{{{\rm{B}}}}}}{{k}_{{{{\rm{I}}}}}}\frac{{\eta }_{I}}{{\eta }_{B}}$$

To test the modeling assumptions (Fig. 4c), the probes were binned by the predicted *K*_D,*i*_, and, for each bin, the observed and predicted binding probabilities were computed using

39 $$p({{{\rm{B}}}}| i)=\frac{E[{k}_{i,{{{\rm{B}}}}}]}{E[{k}_{i,{{{\rm{I}}}}}]}\frac{{\eta }_{I}}{{\eta }_{B}}$$

Here, *E*[*k*_*i*,B_] and *E*[*k*_*i*,I_] were evaluated using the observed and predicted read counts in each bin.

#### Simulations

To test the theoretical consistency of the *K*_D_-seq, we developed simulations of the assay and analyzed the resulting reads with ProBound to see if the ‘ground truth’ parameters used in the simulations were recovered. In a first set of simulations, we computed the binding equilibrium for different TF and DNA library concentrations to test the theoretical consistency and robustness of our approach. A major goal of these simulations was to see if *K*_D_-seq suffers from being in the ‘titration regime’^62^. For single-ligand binding experiments, the titration regime occurs when the concentration of the constant fraction (for example, the DNA probes) greatly exceeds the dissociation constant of the interaction; in this regime, most of the varied fraction (for example, the TF) will be bound until the total concentration of the varied fraction exceeds that of the constant fraction. The resulting quick change in the (unobserved) free concentration makes extraction of accurate *K*_D_ values challenging. We, thus, wondered if this phenomenon impacts *K*_D_-seq, which uses a library of randomized (mostly low-affinity) DNA probes.

To simulate this, we first enumerated all 10-bp DNA probe sequences and computed the *K*_D_ values of these using the binding model for Dll shown in Fig. 4b as the ground truth. To model the coupled binding equilibrium, we first estimated the initial probe frequencies ${[{{{{\rm{DNA}}}}}_{i}]}_{{{{\rm{I}}}}}$ by matching the base frequencies to those observed in the input library (28.8% A, 26.5% C, 14.4% G and 30.3% T) and then used the secant method to find the root of

40 $${[{{{\rm{P}}}}]}_{{{{\rm{F}}}}}={[{{{\rm{P}}}}]}_{{{{\rm{T}}}}}-\mathop{\sum}\limits_{i}{[{{{{\rm{DNA}}}}}_{i}]}_{{{{\rm{I}}}}}\frac{{[{{{\rm{P}}}}]}_{{{{\rm{F}}}}}}{{[{{{\rm{P}}}}]}_{{{{\rm{F}}}}}+{K}_{{{{\rm{D}}}},i}},$$

and finally used the resulting value of [P]_F_ combined with equations (29) and (30) to compute the relative concentrations of all probes in the input, bound and free libraries. Then, 10^6^ sequences were sampled for each library using the multinomial distribution, and ProBound was finally used to learn a *K*_D_-model. This procedure was repeated for all combinations of [P]_T_ and [DNA]_I_ used in Fig. 4e. As expected, the fraction of bound TF molecules increased with DNA concentration (ranging between 0.2–1.1%, 1.0–5.5% and 4.8–24% in the simulations with 20 nM, 100 nM and 500 nM (Extended Data Fig. 8c)). Thus, although both the TF and total DNA concentrations exceed the *K*_D_ for the strongest sequence, the concentration of such probes is very low (because a large majority of probes have low affinity; Extended Data Fig. 8b), and the titration regime can generally be avoided (also see ‘Practical guidelines’ below). Finally, the inferred *K*_D_ values were very close to those predicted by the ground truth model (Fig. 8e), demonstrating the theoretical consistency of our approach.

In a second set of simulations, we investigated how slow binding kinetics of high-affinity probes might impact the final *K*_D_ model. To this end, we modeled the binding kinetics of the library using

41 $${\partial }_{t}{[{{{{\rm{DNA}}}}}_{i}]}_{{{{\rm{B}}}}}={k}_{{{{\rm{on}}}},i}{[{{{\rm{P}}}}]}_{{{{\rm{F}}}}}{[{{{{\rm{DNA}}}}}_{i}]}_{{{{\rm{F}}}}}-{k}_{{{{\rm{off}}}},i}{[{{{{\rm{DNA}}}}}_{i}]}_{{{{\rm{B}}}}}$$

where *k*_on,*i*_ and *k*_off,*i*_ are the on-rates and off-rates for probe *i*. Because most protein is free even at equilibrium (see the equilibrium simulation above), we solved this differential equation under the assumption [P]_F_ = [P]_T_, giving

42 $$p(B| i,t)\equiv \frac{{[{{{{\rm{DNA}}}}}_{i}]}_{{{{\rm{B}}}}}(t)}{{[{{{{\rm{DNA}}}}}_{i}]}_{{{{\rm{I}}}}}}=\frac{{[{{{\rm{P}}}}]}_{{{{\rm{T}}}}}}{{[{{{\rm{P}}}}]}_{{{{\rm{T}}}}}+{K}_{{{{\rm{D}}}},i}}\left(1-{e}^{-t({k}_{{{{\rm{off}}}},i}+{[{{{\rm{P}}}}]}_{{{{\rm{T}}}}}{k}_{{{{\rm{on}}}},i})}\right)$$

To simulate the scenario where high-affinity probes have the slowest kinetics, we assumed that *k*_on_ is diffusion limited (and, thus, sequence independent) and that the sequence specificity is driven by variation in *k*_o*ff*_. After expressing *k*_off,*i*_ in terms of the value for the highest-affinity sequence,

43 $${k}_{{{{\rm{off}}}},i}={k}_{{{{\rm{off}}}},\min }\frac{{K}_{{{{\rm{D}}}},i}}{{K}_{{{{\rm{D}}}},\min }},$$

the binding probability becomes:

44 $$p(B| i,t)=\frac{{[{{{\rm{P}}}}]}_{{{{\rm{T}}}}}}{{[{{{\rm{P}}}}]}_{{{{\rm{T}}}}}+{K}_{{{{\rm{D}}}},i}}\left(1-{e}^{-{k}_{{{{\rm{off}}}},\min }t({K}_{{{{\rm{D}}}},i}+{[{{{\rm{P}}}}]}_{{{{\rm{T}}}}})/{K}_{{{{\rm{D}}}},\min }}\right)$$

Note that this probability only depends on *k*_on_ and *k*_off_ through *K*_D_, which is known, and ${k}_{{{{\rm{off}}}},\min }$. To test how robust *K*_D_-seq is to the value of the latter, we simulated experiments with ${k}_{{{{\rm{off}}}},\min }t\in \left\{0.001,0.01,0.1\right\}$ (Extended Data Fig. 8f), analyzed the resulting reads using ProBound and compared the final *K*_D_ model to the ground truth parameters used in the simulation (Extended Data Fig. 8g). This showed that the true model was recovered for $t\ge 0.1{k}_{{{{\rm{off}}}},\min }^{-1}$, with even shorter incubation times being acceptable at high protein concentrations.

#### Experimental protocol

6×His tagged *Drosophila* Dll protein lacking amino acids N terminal to its homeodomain (DllΔN) was purified by standard procedures. Next, 0.05% Tween 20 was included in the lysis buffer and in the elution buffer to prevent the target protein from sticking to plasticware. The purified protein was quantified by Bradford assay, using BSA as the standard. The 10mer R0 library was generated by annealing the library oligo (GTTCAGAGTTCTACAGTCCGACCTGG-10N-CCAGGACTCGGACCTGGACTAGG) and the SELEX-R primer (CCTAGTCCAGGTCCGAGT), followed by a Klenow-mediated primer extension reaction. The library DNA was purified using Qiagen minElute columns and was quantified using NanoDrop. The SELEX procedure was largely the same as previously described^8^, except that a Cy5-labeled DNA probe, instead of a P32-labeled probe, was used as the marker to indicate where the bound and unbound fractions were. The Cy5-labeled DNA probe was generated by annealing a Cy5-labeled primer to a DNA probe with the desired DNA sequence, followed by Klenow reaction. EDTA was used to stop the reaction. The probe was directly used in the binding reaction, without further purification.

For each SELEX condition, 15 μl of protein solution (at 2× final concentration) in dialysis buffer (20 mM HEPES pH 8.0, 200 mM NaCl, 10% glycerol, 2 mM MgCl_2_, 0.05% Tween 20) was made. The library mixture was made by adding desired amount of the R0 library to 6 μl of 5× binding buffer (50 mM Tris-HCl pH 7.5, 250 mM NaCl, 5 mM MgCl_2_, 20% glycerol, 2.5 mM DTT, 2.5 mM EDTA, 125 ng μl^−1^ of polydIdC, 100 ng μl^−1^ of BSA, 0.125% Tween 20) and filling to 15 μl with water. The protein and DNA parts were mixed and incubated at room temperature for 30–40 minutes before loading the gel. For Cy5-labeled markers, 15 μl of 200 nM DllΔN in dialysis buffer was mixed to 15 μl of DNA mixture (6 μl of 5× binding buffer, 8 μl of water and 1 μl of 200 nM probe) and incubated at room temperature for 30–40 minutes.

After running the gel, gel slices corresponding to the bound and unbound fractions were cut from the gel and were each place in a 500-μl tube with several needle poked holes at the bottom. The 500-μl tubes were each placed within a 2-ml tube and spun at maximum speed at room temperature to smash the gel. Then, 650 μl of DNA extraction buffer (10 mM Tris-HCl, pH 7.5, 150 mM NaCl, 1 mM MgCl_2_, 0.5 mM EDTA, pH 8.0) and 50 μl of 20% SDS were added to each smashed gel sample, and the tubes were rotated at room temperature for 2–4 hours. The tubes were then spun at maximum speed at room temperature for 2 minutes. Then, 650 μl of sample was transferred to a Spin-X filter column and spun at room temperature at the maximum speed for 2 minutes. The DNA in flow-through was purified by phenol chloroform extraction, followed by isopropanol precipitation. Then, 20 μg of glycogen was used to facilitate precipitation, and the DNA pellet was dissolved in 20 μl of Qiagen EB buffer.

Each purified SELEX DNA was properly diluted such that the following PCR program gave good library yield for all samples. The one-step library preparation was done in a 50-μl reaction, which contains 5 μl of properly diluted SELEX DNA, 10 nM of one of the eight SELEX-for primers, 10 nM of the common SELEX-rev primer, 1 μM of NEB universal primer for Illumina and 1 μM of selected NEB index primer for Illumina. PCR was done with the Phusion DNA polymerase (NEB), using the following program: one cycle of 98 °C for 30 seconds; five cycles of 98 °C for 10 seconds, 60 °C for 30 seconds and 72 °C for 15 seconds; ten cycles of 98 °C for 10 seconds and 65 °C for 75 seconds; one cycle of 65 °C for 5 minutes; and hold at 4 °C. Amplified libraries were purified using 1.5 volume (75 μl) of AMPure beads and eluted with 15 μl of Qiagen EB buffer. The libraries were pooled and sequenced using Illumina NextSeq 550, following standard procedures. The forward primers consisted of left and right constant sequences (ACACTCTTTCCCTACACGACGCTCTTCCGATCT- and -GTTCAGAGTTCTACAGTCCGA, respectively), flanking a library-specific barcode: 1) --, 2) -AGAC-, 3) -TCAGAC-, 4) -CAGAC-, 5) -C-, 6) -GAC-, 7) -AC- and 8) -TTCAGAC-. In addition, we used the reverse primer GACTGGAGTTCAGACGTGTGCTCTTCCGATCT-CCTAGTCCAGGTCCGAGT, the NEB universal primer AATGATACGGCGACCACCGAGATCTACACTCTTTCCCTA-CACGACGCTCTTCCGATCT and the NEB index primer CAAGCAGAAGACGGCATACGAGAT-[6bp index]-GTGACTGGAGTTCAGACGTGTGCTCTTCCGATCT.

#### EMSA validation

The same batch of the DllΔN protein that was used in the SELEX experiments was also used in the measurement of the absolute *K*_*D*_ values of DllΔN to selected DNA sequences. The EMSA experiments were performed following regular protocol. In brief, the protein was diluted with dialysis buffer to 2× of the desired final concentration in a total volume of 15 μl. The DNA mixture was made by mixing 6 μl of 5× binding buffer, 8 μl of water and 1 μl of 200 nM Cy5-labeled DNA probe. The DNA probes had the same flanks as the 10mer SELEX library and the indicated middle 10 bp. The protein part and the DNA part were mixed well (giving a final DNA probe concentration of 6.7 nM) and incubated at room temperature for 30–40 minutes before loading the 0.5× native TBE gel.

After running the gel, an image was taken using the Typhoon imager, and the band intensity was quantified using Fiji version 1.52n (Supplementary Table 5). In brief, each band was selected using the rectangle selection tool, and the selected regions were converted to histograms. A straight line was drawn at the bottom of each histogram, and the areas of the enclosed peak regions were quantified and used as band intensity.

For each probe, *K*_D_ was finally estimated by fitting the binding probability

45 $$\begin{array}{l}p({{{\rm{B}}}};\,{[{{{\rm{P}}}}]}_{{{{\rm{T}}}},a})\\={\left(1+\frac{2{K}_{{{{\rm{D}}}}}}{{[{{{\rm{P}}}}]}_{{{{\rm{T}}}},a}-{[{{{\rm{DNA}}}}]}_{{{{\rm{T}}}}}-{K}_{{{{\rm{D}}}}}+\sqrt{{({[{{{\rm{P}}}}]}_{{{{\rm{T}}}},a}-{[{{{\rm{DNA}}}}]}_{{{{\rm{T}}}}}-{K}_{{{{\rm{D}}}}})}^{2}+4{K}_{{{{\rm{D}}}}}{[{{{\rm{P}}}}]}_{{{{\rm{T}}}},a}}}\right)}^{-1},\end{array}$$

where [P]_T,*a*_ is the total TF concentration in band *a*, and [DNA]_T_ is the total DNA concentration, to the quantitated intensities *y*_B,*a*_ and *y*_F,*a*_ of the bound and free bands, respectively (Supplementary Table 5). Specifically, after introducing the band-specific intensity scaling factors *α*_B_ and *α*_F_, we found the parameters that minimized the loss function

46 $$\begin{array}{l}({K}_{{{{\rm{D}}}}},{\alpha }_{{{{\rm{B}}}}},{\alpha }_{{{{\rm{F}}}}})\\={\sum }_{a}\left[{\left(p({{{\rm{B}}}};\,{[{{{\rm{P}}}}]}_{{{{\rm{T}}}},a})-{\alpha }_{{{{\rm{B}}}}}{y}_{{{{\rm{B}}}},a}\right)}^{2}+{\left((1-p({{{\rm{B}}}};{[{{{\rm{P}}}}]}_{{{{\rm{T}}}},a}))-{\alpha }_{{{{\rm{F}}}}}{y}_{{{{\rm{F}}}},a}\right)}^{2}\right].\end{array}$$

#### Practical guidelines

As with any assay, *K*_D_-seq can produce inaccurate measurements given unsuitable experimental conditions. One strength of *K*_D_-seq is that many such conditions can be diagnosed computationally. Below are practical guidelines for designing successful *K*_D_-seq experiments and for detecting problems, should they occur.

**Robust probe depletion in the free library**. For a *K*_D_-seq experiment to be successful, ProBound needs to estimate the net fraction of bound DNA *p*(B). Intuitively, ProBound accomplishes this by separately computing the relative probe frequencies in the input, bound and free libraries and then finding the value of *p*(B) that makes the relative frequencies satisfy the sum rule in equation (32) (technically, ProBound maximizes the likelihood of the full model, as detailed above). For this estimate to be robust, is important that some high-affinity probes have detectable depletion in the free library; otherwise, the input and free libraries are identical, and the sum rule is satisfied for *p*(B) = 0. This estimate becomes less robust in two experimental regimes. First, no probe will be depleted if the TF concentration is well below the *K*_D_ of the strongest probe. Second, the depletion signal in the free library is reduced when [DNA]_I_ ≫ [P]_T_ because, at most, a small fraction of the library can be bound in this regime. An example of the latter is the experiment with 500 nM DNA and 100 nM TF, where only 2% of the library was bound. Computationally, low depletion in the free library is most easily detected using the enrichment plots in Fig. 4c.

**Robust estimate of relative binding affinities**. ProBound estimates relative *K*_D_ values using both probe enrichment in the bound library and probe depletion in the free library. Thus, although saturation compresses the relative selection for high-affinity probes in the bound library (because all saturated probes have *P*(B∣*i*) ≈ 1), relative *K*_D_ values can still be estimated because the saturated probes differ in depletion in the free library. However, because the number of reads corresponding to high-affinity probes decreases as these probes become increasingly saturated, excessive saturation (that is, ${[{{{\rm{P}}}}]}_{{{{\rm{T}}}}}\gg {\min }_{i}{K}_{{{{\rm{D}}}},i}$) tends to make the *K*_D_ estimates for the highest-affinity probes less robust. Examples of this include the experiments with 3,300 nM Dll in Fig. 4e. Excessive saturation is most easily detected using the enrichment plots in Fig. 4c.

**Avoiding the titration regime**. As discussed above, single-ligand *K*_D_ measurements can be compromised when conducted in the ‘titration regime’^62^; if *K*_D_ is much smaller than the ligand concentration (assuming this is the constant fraction), *K*_D_ no longer corresponds to the protein concentration at which 50% of ligands are bound but must, rather, be estimated through non-linear curve fitting that models titration to estimate the free protein concentration. However, such curve fitting becomes increasingly error-prone as the ligand concentration increases. This regime should generally be avoided.

However, *K*_D_-seq has two advantages compared to single-ligand experiments: First, the vast majority of ligands have low affinity (see simulation above), and the concentration of high-affinity ligands is, therefore, much lower than the total ligand concentration. Thus, titration can be avoided even when the total library concentration substantially exceeds the smallest *K*_D_ in the library. Second, ProBound estimates the fraction of ligands bound, which, in turn, can be used to estimate the fraction of protein bound (Equation (34)). This provides an internal measure to monitor titration effects. If more than 5–10% of the TF molecules are estimated to be bound (for example, experiment with 500 nM library and 100 nM Dll in Fig. 4e), the assay should be repeated with decreased library concentration.

**Binding equilibrium**. For *K*_D_ measurements to be accurate, it is important that the binding reaction has reached equilibrium^62^. In particular, high-affinity probes can have a low off-rate and, thus, take longer time to reach equilibrium. However, our simulations above indicated that *K*_D_-seq produces stable binding models after 10% of the naively expected equilibrium time (based on the off-rate for the highest-affinity probe). To understand this, note that Equation (42) can be used to express the equilibration time *t*_eq,*i*_ for probe *i* as

47 $${t}_{{{{\rm{eq}}}},i}={k}_{{{{\rm{off}}}},i}^{-1}\frac{1}{1+{[{{{\rm{P}}}}]}_{{{{\rm{T}}}}}/{K}_{{{{\rm{D}}}},i}}$$

We, thus, see that saturated probes, which have [P]_T_/*K*_D,*i*_ > 1, reach binding equilibrium faster than naively expected given ${k}_{{{{\rm{off}}}},i}^{-1}$. This observation, combined with the experimental constraint that high-affinity probes should be at least moderately saturated (see above), explains the relative robustness of *K*_D_-seq with regard to incubation time. Nonetheless, when working with systems for which the off-rates are unknown, it is advisable to repeat the assay for multiple incubation times to validate that equilibrium has been reached.

**Validating the binding curve**. Although ProBound can estimate *K*_*D*_ values using binding data for a single protein concentration, the method assumes that the binding probability follows Equation (29). However, deviations from this binding curve can occur—for example, due to cooperative binding at high protein concentrations. When characterizing a new protein, it can, therefore, be prudent to validate the binding curve by repeating the assay for multiple protein concentrations.

**Multi-concentration input-versus-bound experiments**. ProBound can learn a *K*_D_ model by jointly analyzing the input and bound libraries of SELEX experiments conducted at different protein concentrations (Extended Data Fig. 7d). Intuitively, this approach uses low-concentration libraries (which ideally have a linear affinity-versus-binding relationship) to learn the relative binding affinities and high-concentration libraries (which should have saturated high-affinity probes) to determine the affinity scale. Although limited saturation of high-affinity probes in the lowest-concentration library can be acceptable as long as the relative-affinity model (which then mainly is constrained by the non-saturated lower-affinity probes) generalizes to the highest-affinity probes, such saturation should be avoided if possible. This effect may explain the slightly lower dissociation constant estimated in Extended Data Fig. 7d (which uses input/bound) compared to Extended Data Fig. 7c (which also uses the free library).

### Peak-free motif discovery from ChIP-seq data

#### ProBound analysis

To analyze the GR ChIP-seq data from the IMR90 cell line^47^, we first aligned the (single-end) Input and ChIP reads to the genome and extracted a sufficiently long (200-bp) sequence downstream of the ${5}^{\prime}$-end genomic position of the mapped read. Next, we randomly sampled 10^6^ reads from each library and constructed a count table containing the Input and ChIP read counts in the first and second columns, respectively. ProBound was then configured to model this table as a single-round SELEX experiment. Because GR binds DNA as a homodimer, we configured ProBound to impose reverse-complement symmetry while fitting free-energy parameters for the primary motif. We then iteratively added three additional binding modes to the model to capture the influence of potential co-factors. To analyze the GR ChIP-seq data from the murine hippocampus^51^, we followed a similar procedure and constructed one count table for each of the three CORT concentrations (sampling 10^5^ sequences per library) and then configured ProBound to jointly model all count tables using a single reverse-complement-symmetric binding mode.

#### Other methods

Raw FASTQ files corresponding to the IMR90 GR ChIP and Input sequences from Starick et al.^47^ were downloaded from the European Nucleotide Archive using accession number PRJEB7372. SAM files of the input and ChIP sequences were created by aligning to the hg19 genome using bowtie2 (version 2.4.4) with default settings.

**HOMER**: HOMER (version 4.11.1)^63^ with default settings was used to analyze the SAM files; ‘tag directories’ for both the ChIP and Input sequences were first created using makeTagDirectory. Next, the command analyzeChIP-Seq.pl Tagged_GR_ChIP/ hg19 -i Tagged_GR_Input/ was executed to infer binding motifs.

**MEME-ChIP**: MACS2 (version 2.2.7.1)^64^ with default settings was used to discover enriched peak regions. Then, 500-bp genomic regions—250 bp upstream and downstream of the discovered peak centers—were extracted from the resulting BED files using bedtools. The MEME-ChIP webserver was used to analyze these sequences with default settings and the ‘Look for palindromes only’ option selected.

**NoPeak**: The NoPeak repository^65^ was downloaded from GitHub, and the SAM files were converted to BED files following the example in the repository: samtools view -bS GR_chip.sam ∣ bedtools bamtobed ∣ sort -k1,1 -k2n > GR_chip.bed.

These BED files were analyzed using NoPeak with default settings (kmer length = 8). This required 128 GB of RAM to complete; other kmer lengths were tried (>8) but failed as NoPeak ran out of memory.

### Kinase-seq

#### ProBound analysis

In this assay, a library of peptide substrates *S*_*i*_ is treated with a enzyme *E*, and the concentrations of the products *P*_*i*_ are quantified using high-throughput sequencing (see below). This reaction can be modeled using Michaelis–Menten kinetics generalized to multiple substrates:

48 $$E+{S}_{i}\mathop{\rightleftharpoons }\limits^{{k}_{{{{\rm{on}}}},i}}_{{k}_{{{{\rm{off}}}},i}}E:{S}_{i}\mathop{\to }\limits_{{k}_{{{{\rm{cat}}}},i}}E+{P}_{i}$$

In the limit of low enzyme concentration, the reaction quickly reaches a quasi-steady state with

49 $$[E:{S}_{i}]=[E][{S}_{i}]/{K}_{{{{\rm{M}}}},i}$$

where *K*_M,*i*_ = (*k*_off_ + *k*_cat,*i*_) / *k*_on,*i*_ is the Michaelis constant for substrate *i*. In this limit, the change in substrate concentration is given by

50 $${\partial }_{t}[{S}_{i}]=-{k}_{{{{\rm{eff}}}},i}[{S}_{i}][E]$$

where *k*_eff,*i*_ = *k*_cat,*i*_ / *K*_M,*i*_ is the catalytic efficiency. Integrating this equation yields

51 $$[{S}_{i}](t)=[{S}_{i}](0){e}^{-{k}_{{{{\rm{eff}}}},i}\int\nolimits_{0}^{t}[E](t')dt'}$$

where [*S*_*i*_](0) is the substrate concentration right after the quasi-equilibrium was reached. The concentrations in the product library can then be expressed as

52 $$[{P}_{i}](t)={[{S}_{i}]}_{{{{\rm{total}}}}}\left(1-\frac{1+[E](t)/{K}_{{{{\rm{M}}}},i}}{1+[E](0)/{K}_{{{{\rm{M}}}},i}}{e}^{-{k}_{{{{\rm{eff}}}},i}\overline{E}(t)t}\right)$$

where ${[{S}_{i}]}_{{{{\rm{total}}}}}=[{S}_{i}]+[E:{S}_{i}]+[{P}_{i}]$ is concentration in the initial library, and $\overline{E}(t)={t}^{-1}\int\nolimits_{0}^{t}[E](t')dt'$ is the time-averaged enzyme concentration. This can be simplified further by noting that only a small fraction of substrates are bound in the limit of low enzyme concentration

53 $$[E:{S}_{i}]/[{S}_{i}]=[E]/{K}_{{{{\rm{M}}}},i}\ll 1$$

and, thus,

54 $$[{P}_{i}](t)={[{S}_{i}]}_{{{{\rm{total}}}}}\left(1-{e}^{-{k}_{{{{\rm{eff}}}},i}\overline{E}(t)t}\right)$$

Note that the selection only differs between probes through *k*_eff,*i*_. ProBound can, thus, model the assay using Eq. (8) with *δ*→−*∞* and

55 $${Z}_{{{{\rm{bound}}}},i,P}={k}_{{{{\rm{eff}}}},i}\overline{E}(t)t$$

Here, $\overline{E}(t)$ depends on both *K*_D,*i*_ and [*S*_*i*_] throughout the reaction and is generally unknown. We here assume that most enzyme is free so that $\overline{E}(t)={[E]}_{{{{\rm{total}}}}}$; a lower (free) enzyme concentration would lead to a global rescaling of *k*_eff,*i*_ but not affect the relative efficiency or its sequence dependence.

#### Preparation of degenerate peptide library to profile tyrosine kinase specificity

The degenerate peptide library contained 11 residue sequences with five randomized amino acids flanking either side of a fixed central tyrosine residue. These sequences were fused to the eCPX bacterial surface display scaffold^66^. To clone this library, we first amplified the eCPX-coding sequence with a $3^{\prime}$ SfiI restriction site. This was fused to the random library in another PCR step using the following degenerate oligonucleotide: GCTGGCCAGTCTGGCCAG-NNSNNSNNSNNSNNStatNNSNNSNNSNNSNNS-GGAGGGCAGTCTGGGCAGTCTG, which contains a 5′ SfiI site. The resulting amplified product was digested with SfiI restriction endonuclease, purified and ligated into the SfiI-digested pBAD33-eCPX plasmid, as described previously^53^. The ligation reaction was concentrated and desalted and then used to transform DH5*α* cells by electroporation. Transformed cells were grown overnight in liquid culture, and then the plasmid DNA library was extracted and purified using a commercial Midiprep kit.

#### Preparation of biotinylated antibody

The phosphotyrosine monoclonal antibody (pY20, conjugated to the fluorophore, perCP-eFluor 710, Invitrogen, cat. no. 46-5001-42) was desthiobiotinylated before use in the specificity screen. The antibody was first purified away from BSA and gelatin by anion exchange using a salt gradient of 0 M NaCl to 1 M NaCl in 0.1 M potassium phosphate buffer. The fractions that eluted after 0.2 M NaCl were pooled and then buffer-exchanged into 0.1 M potassium phosphate by dilution and centrifugal filtration. The antibody was then labeled in a 200-μl small-scale reaction using the DSB-X labeling kit (Molecular Probes) according to the manufacturer’s instructions. Concentration of the antibody was monitored by its absorbance at 490 nm to determine percentage yield. The average final concentration of the antibody was around 0.2 mg ml^−1^. The specificity of the antibody was validated using cells expressing displayed peptides. Cells treated with a tyrosine kinase without ATP show no background antibody staining. By contrast, cells expressing displayed peptides, treated with tyrosine kinase and 1 mM ATP, show increasing antibody staining as a function of phosphorylation time.

#### High-throughput specificity screen

The catalytic domain of the human tyrosine kinase c-Src was screened against the degenerate peptide library as described previously^53^—one main difference being the use of magnetic beads to isolate phosphorylated cells rather than fluorescence-activated cell sorting. In short, *Escherichia coli* MC1061 cells transformed with the library were grown to an optical density of 0.5 at 600 nm. Expression of the surface-displayed peptides was induced with 0.4% arabinose for 4 hours at 25 °C. After expression, the cell pellets were collected and subject to a wash in PBS. Phosphorylation reactions of the library were conducted with 500 nM of purified c-Src and 1 mM ATP in a buffer containing 50 mM Tris, pH 7.5, 150 mM NaCl, 5 mM MgCl_2_, 1 mM TCEP and 2 mM sodium orthovanadate. Time points were taken at 5 minutes, 20 minutes and 60 minutes. Kinase activity was quenched with 25 mM EDTA, and the cells were washed with PBS. Kinase-treated cells were labeled with roughly 0.05 mg ml^−1^ of the biotinylated pY20 antibody for 1 hour and then washed again with PBS containing 0.2% BSA.

The phosphorylated cells were isolated with Dynabeads FlowComp Flexi (Invitrogen) following the manufacturer’s protocol. In total, two populations were collected for each time point: cells that did not bind to the magnetic beads and eluted after each wash (unbound) and cells that bound to the magnetic beads and eluted after the addition of the release buffer (bound). After isolation of these two populations, the cell pellet was collected, resuspended in water and then lysed by boiling at 100 °C for 10 minutes. The supernatant from this lysate was then used as a template in a 50-μl PCR reaction to amplify the peptide codon DNA sequence using the same forward and reverse TruSeq-eCPX primers as described previously^53^. The product of this PCR reaction was then used as a template for a second PCR reaction to append unique 5′ and 3′ indices. The resulting PCR products were purified by gel extraction, and the concentration of each sample was determined using QuantiFluor dsDNA System (Promega). Each sample was pooled to equal molarity and sequenced by paired-end Illumina sequencing on a MiSeq instrument. The deep sequencing data were processed as described previously^53,67^. The paired-end reads were merged using FLASH (version FLASH2-2.2.00)^68^, and the adapter sequences were trimmed using the software Cutadapt (version 3.5)^69^. The remaining sequences were translated into amino acid codes, and sequences containing stop codons were removed.

#### Validation measurement of phosphorylation rates

To validate predictions made by ProBound, phosphorylation rates were determined in vitro using purified c-Src and 11 synthetic peptides (purchased from Synpeptide). The phosphorylation reactions were carried out at 37 °C using 500 nM purified c-Src and 100 μM peptide in a buffer containing 50 mM Tris, pH 7.5, 150 mM NaCl, 5 mM MgCl_2_, 1 mM TCEP and 2 mM sodium orthovanadate. Reactions were initiated by the addition of 1 mM ATP, and, at various time points, 100 μl of the solution was quenched with 25 mM EDTA (every 10 seconds for the faster reactions, every 2–10 minutes for the slower reactions). Each reaction was carried out in triplicate.

The concentration of the substrate and the phosphorylated product at each time point was determined by reversed-phase HPLC with UV detection at 214 nm (Agilent 1260 Infinity II). A 40-μl volume of the quenched reaction was injected onto a C18 column (ZORBAX 300SB-C18, 5 μm, 4.6 × 150 mm). A gradient system was used with solvent A (water and 0.1% TFA) and solvent B (acetonitrile and 0.1% TFA). Elution of the peptides was performed at a flow rate of 1 ml min^−1^ using the following gradient: 0–2 minutes: 5% B; 2–12 minutes: 5–95% B; 12–13 minutes: 95% B; 13–14 minutes: 95–5% B; and 14–17 minutes: 5% B. The peak areas of the substrate and product were calculated using Agilent OpenLAB ChemStation software (version C.01.09). The initial rate for each peptide was obtained by fitting a straight line to a graph of peak area as a function of time in the linear regime of the reaction progress curve and calculating the slope of the line.

### Reporting summary

Further information on research design is available in the Nature Research Reporting Summary linked to this article.

## Online content

Any methods, additional references, Nature Research reporting summaries, source data, extended data, supplementary information, acknowledgements, peer review information; details of author contributions and competing interests; and statements of data and code availability are available at 10.1038/s41587-022-01307-0.

## Supplementary information

Supplementary Information Software Manual and Description of Configuration Files

Reporting Summary

Supplementary Tables Training Database, HPLC Validation, SELEX Experiments, ChIP+DNA+DNAse GLM and EMSA Validation

## Data Availability

The sequencing data generated during the current study have been deposited in the Gene Expression Omnibus under accession number GSE175942. Source data for Figs. 4d and 6d are provided in Supplementary Tables 2 and 5.

## Code availability

TF binding models and software for using them can be accessed at motifcentral.org. The ProBound software and a dedicated compute server for running ProBound are available at probound.bussemakerlab.org.
