# Supplementary material for: Prediction of protein–ligand binding affinity from sequencing data with interpretable machine learning
Source: Nat Biotechnol. 2022 May 23;40(10):1520–7. doi: 10.1038/s41587-022-01307-0 (PMC9546773; doi:10.1038/s41587-022-01307-0)
Supplement: Supplementary file 1 — Software Manual and Description of Configuration Files [file 41587_2022_1307_MOESM1_ESM.pdf]

nature portfolio
https://doi.org/10.1038/s41587-022-01307-0

## Supplementary information

# Prediction of protein–ligand binding affinity from sequencing data with interpretable machine learning

In the format provided by the
authors and unedited

# EXTENDED DATA METHODS

## Software Manual

ProBound is written in Java and is easiest run on our dedicated compute server:

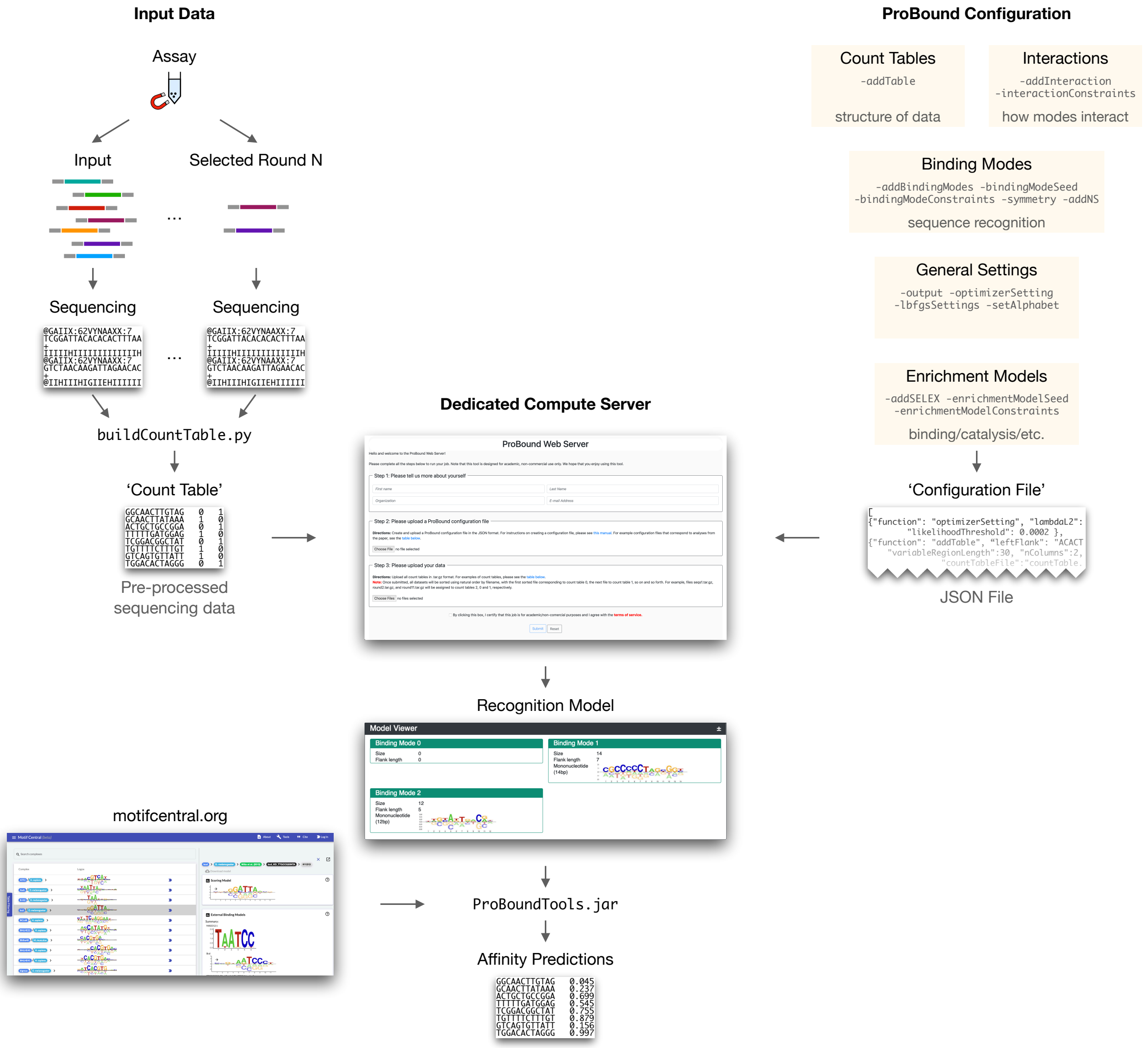


This server, which can be accessed for academic use via probound.bussemakerlab.org, takes two types of files as input: a single JSON-formatted configuration file and one or more 'count tables' (see below). Upon completion, the server returns a webpage containing sequence logos and a JSON-formatted model file (see figure above). This model file is in the same format as those found on MotifCentral.org, our resource of high-quality pre-computed ProBound models used in this study. These JSON-formatted model files can be used by `ProBoundTools.jar`, a lightweight Java tool for computing affinities for arbitrary sequences. `ProBoundTools.jar` is available both on MotifCentral and at github.com/BussemakerLab.

Count tables are tab-separated text files (TSVs) where every row contains the sequence and count of every observed probe in the library (i.e. the number of times each library sequence was observed in the data) as it progresses through the various selection rounds in a specific assay. Examples of count table layouts for some of the assay designs in this paper are shown in the table below. It should be pointed out that any integrative analyses – such as the creation of consensus binding models, co-factor interaction models or methylation-aware binding models – require multiple count tables corresponding to individual assays. For instance, constructing meCpG methylation-aware binding models requires two count tables: one for the methylated selection round and one for the unmethylated selection round. To make it easier to generate count tables from raw sequencing data, we have provided an easy-to-use Python script called `buildCountTable.py`. This script, which is included in the GitHub repository, takes sequence lists as input and returns correctly formatted count tables that can be used by the compute server.

| Assay Design | Column Labels |
|---|---|
| Single-round SELEX | `seq input round1` |
| Multi-round SELEX | `seq input round1 round2 round3` |
| $K_D$-seq | `seq input round1_bound round1_unbound` |
| ChIP-seq | `seq input chip` |
| EpiSELEX-seq **methylated** | `seq input round1` |
| EpiSELEX-seq **unmethylated** | `seq input round1` |
| Kinase-seq | `seq input 5min 20min 60min` |

**Table 1.** Count table layouts.

The JSON-formatted configuration file contains commands, called functions, that tell ProBound how the input data is structured and how to configure the various model components (i.e. how many binding modes to use, whether or not the binding modes should interact, the kind of selection process that generated the data, etc.). Functions can have attributes that further define these settings, and the configuration file conforms to the following template:

```
 [
  { "function": "functionName1", "attribute1": "value1", ...},
  { "function": "functionName2", "attribute1": "value1", ...},
  ...
]
```

The remainder of the document contains detailed descriptions of all the configuration file functions and their attributes, the structure of the JSON-formatted output file, and the configuration files that were used for the fits presented in the main text.

## Configuration File Functions

The functions can be grouped into five categories:

### *General Settings*

**`output`** Function that specifies where and how the output should be written

- `outputPath` (string, required): Path to the output directory
- `baseName` (string, required): String appended to the beginning of all output files
- `printTrajectory` (boolean, default is `false`): Should the optimizer trajectory be saved?
- `verbose` (boolean, default is `false`): Should the message output to `STDOUT` be verbose?

**`optimizerSetting`** Function that specifies optimization parameters

- `lambdaL2` (float, default is `1-e7`): Value of weight $\lambda$ of the $L_2$ regularizer
- `pseudocount` (float, default is `0`): Value of weight $k_{\text{Dirichlet}}$ of the Dirichlet regularizer
- `expBound` (float, default is `40`): Value of weight $\theta_{\text{max}}$ of the exponential barrier regularizer
- `nThreads` (integer, default is `4`): Number of threads to use
- `nRetries` (integer, default is `3`): Max retries before the optimizer proceeds to the next step
- `likelihoodThreshold` (integer, default is `0`): Smallest improvement in likelihood required for a model variation to be accepted

**`lbfgsSettings`** Function that specifies options for the L-BFGS optimizer

- `memory` (integer, default is `100`): Number of previous steps kept in memory
- `maxIters` (integer, default is `500`: Maximum iterations

- convergence (float, default is 1e-7): Convergence criteria

**setAlphabet** Function that specifies the alphabet

- letterOrder (string, default is ACGT): String defining the set of valid letters and their order
- letterComplement (string, default is "C-G,A-T"): String defining what letters are mapped to each other by the complementarity transformation. The two letters in a pair are connected by dashes and pairs are separated by commas.

### *Count Tables*

**addTable** Function that defines the input count tables

- countTableFile (string, required): Path to the count table file. The table should be tab separated, have the variable region of the probe sequences in the first columns, and have the number of occurrences of each probe in each SELEX library in the following columns. This file can be gzipped. All sequences must have equal length.
- inputFileType (string, default is tsv): Format of the input file. Either tsv or tsv.gz.
- nColumns (integer, required): Number of columns in the table, ignoring the first one containing probe sequences
- variableRegionLength (integer, required): Length of all probe sequences
- rightFlank (string, default is ""): The constant flanking sequence to the right of the variable region
- leftFlank (string, default is ""): The constant flanking sequence to the left of the variable region
- modeledColumns (list of integers, default is [-1]): Specifies the columns in the count table that should be modeled; [-1] includes all columns.
- transliterate (objects, default is {"in":[], "out":[]}): List of edits that should be made to the probe sequences in order to encode DNA modifications. in lists the probe subsequence that should be substituted and out lists the substitutes. The lists in and out must have equal length, and each pair of sequences must have equal length.

### *Enrichment Models*

**addSELEX** Function that adds an enrichment model to the overall model; these models are associated, one-to-one, with count tables in sequential order.

- modelType (string, default is SELEX): Type of enrichment model, either SELEX, rhoGamma, and ExponentialKinetics
- bindingModes (list of integers, default is [-1]): Defines the binding modes in the enrichment model; [-1] includes all binding modes.
- bindingModeInteractions (list of integers, default is [-1]): Defines the binding mode interactions in the enrichment model; [-1] includes all interactions.
- cumulativeEnrichment (boolean, default is true): Should the enrichment accumulate across columns? (models repeated selection)
- concentration (float, default is 1): Value of the fixed concentration factor used to multiply all activities in the enrichment model
- bindingSaturation (boolean, default is false): For SELEX enrichment models, is the selection linear ($\propto x$ ) or or saturated ($\propto x/(1+x)$)?

**enrichmentModelSeed** Function that defines enrichment model parameter seeds

- rho (list of floats): Vector containing initial $\rho_r$ values for every round $r$ in rhoGamma models

- `gamma` (list of floats): Vector containing initial $\gamma_r$ values for every round $r$ in `rhoGamma` models

**`enrichmentModelConstraints`** Function that defines the constraints on and optimization process of enrichment model parameters

- `fitRho` (boolean, default is `false`): For `rhoGamma` models, should $\rho$ be optimized?
- `fitGamma` (boolean, default is `false`): For `rhoGamma` models, should $\gamma$ be optimized?
- `roundSpecificRho` (boolean, default is `true`): For `rhoGamma` models, can each $\rho_r$ have an independent value?
- `roundSpecificGamma` (boolean, default is `true`): For `rhoGamma` models, can each $\gamma_r$ have an independent values?
- `trySaturation` (boolean, default is `false`): For `SELEX` models, check if setting `bindingSaturation=true` improves the model.

### *Binding modes*

**`addBindingMode`** Function that adds a binding mode and assigns it a running index, starting at 0

- `size` (integer, required): The width of the binding mode
- `flankLength` (integer, default is `0`): Distance into the fixed flanking region that is scored by the binding mode
- `dinucleotideDistance` (integer, default is `0`): Maximum distance between the two letters of the dimer sequence features that are included in the $\Delta\Delta G$ model. `0` inactivates the dimer features, `1` includes only adjacent letters, such as `CG`.
- `singleStrand` (boolean, default is `false`): Only score and include the forward strand in $Z_{\text{bound}}$.
- `positionBias` (boolean, default is `false`): Should the position-bias factor be included ($\omega_a(x)$ optimized) or not ($\omega_a(x) = 1$)?

**`addNS`** Function that adds a non-specific binding mode (shorthand function for adding a mode with `size=0`)

**`bindingModeSeed`** Function that defines the binding mode parameter seeds

- `index` (integer): Index of the binding mode to be seeded; if no index is provided, seeding is applied to all modes
- `mononucleotideIUPAC` (string): Seeds the binding mode to recognize sequences consistent with an IUPAC string. At each position, matches get $\beta_{a,\phi} = 0$ and mismatches get $\beta_{a,\phi} = -1$.
- `mononucleotideString` (string): Seeds the binding mode to recognize sequences consistent with a string. At each position, matches get $\beta_{a,\phi} = 1$ and mismatches give $\beta_{a,\phi} = 0$. The period character (`.`) is a wildcard and matches any letter.

**`bindingModeConstraints`** Function that defines the constraints on and the optimization process of binding mode parameters

- `index, integer, required`: Index of the binding mode that will be manipulated
- `symmetryString` (string, default is `null`): String that defines a symmetry on the binding mode. Two formats are possible:
  - The first format specifies a symmetry by using letters and digits to identify equivalent positions in the binding mode. Upper and lower case letters are related through complement and digits are self-complementary. For example, the string `ab1BA` specifies a reverse-complement symmetric binding mode of size five. Here complementarity relates `a` ↔ `A`, `b` ↔ `B`, and `1` ↔ `1`. The string `ab1BAab1BA` specifies a 10bp binding site with a tetrameric symmetry. The pipe sign (`|`) is a barrier for dinucleotide interactions. This divides the binding mode into regions and removes dinucleotide interactions that connect different regions.

– The second format specifies a sequence of blocks that together fill in the binding mode. Each block is assigned an ID number and two block with the same ID have identical sequence recognition. A block with a negative ID is the reverse complement of a blocks with same but positive ID. Each block can be constrained to be reverse complement symmetric. For example, the symmetry string: `1:6:True` corresponds to a 6bp reverse-complement symmetric block, `1:3:False,1:3:False` corresponds to two concatenated 3bp blocks in head-to-tail configuration, `1:3:False,-1:3:False` corresponds to a two 3bp blocks in the head-to-head configuration. Recognition of dimer sequence features that span blocks are prohibited.

Note that the footprint of a binding mode cannot be modified if a symmetry is specified since the expanded binding mode would no longer have the size specified by the symmetry string.

- `roundSpecificActivity` (boolean, default is `true`): Can binding mode activities have independent values across rounds (count table columns)?
- `experimentSpecificActivity` (boolean, default is `true`): Can binding mode activities have independent values across experiments (count tables)?
- `experimentSpecificPositionBias` (boolean, default is `true`): Can position bias parameters have independent values across experiments? Must be `true` if experiments have different probe lengths.
- `optimizeSize` (boolean, default is `false`): Should the size of the binding mode be optimized? If `true`, the binding mode is (separately) expanded to the left and to the right, the model parameters are re-optimized, and the expanded binding mode is kept if the likelihood improved more than `likelihoodThreshold`.
- `optimizeSizeHeuristic` (boolean, default is `false`): Same as `optimizeSize`, but the binding mode is symmetrically expanded to the left and right and the flank length is incremented
- `optimizeFlankLength` (boolean, default is `false`): Should the flank length be optimized? If `true`, the flank length is incremented, the model parameters are re-optimized, and the new model is kept if the likelihood improved more than `likelihoodThreshold`.
- `optimizeMotifShift` (boolean, default is `false`): Should shifted versions of the binding mode be tested? If `true`, the motif of the binding model is shifted to the left and right (separately), the model parameters are re-optimized, and the new model is kept if the likelihood improved more than `likelihoodThreshold`.
- `optimizeMotifShiftHeuristic` (boolean, default is `false`): Same as `optimizeMotifShift`, but only a single shift is tested. This shift is found by first computing the information content for each position in the binding mode, then computing the 'center of mass' of the information content, and finally computing the shift such that the center of mass is at the center of the binding mode.
- `maxSize` (integer, default is `-1`): Maximum binding mode size;`-1` indicates no limit
- `maxFlankLength` (integer, default is `-1`): Maximum flank length; `-1` indicates no limit
- `informationThreshold` (float, default is `0.1`): Threshold on the information content (computed for the first two and last two bases in the binding mode), determining if `optimizeSize` and `optimizeSizeHeuristic` should attempt to expand the binding mode to the left and right.
- `positionBiasBinWidth` (integer, default is `1`): Configures the set of possible binding configurations in the probe sequence to be partitioned into bins with specified width and constrains the position-bias parameters $\omega_a(x)$ (where $x$ is a configuration) to be constant in each bin, thus reducing the number of independent parameters. By default, each bin contains a single configuration and no constraint is thus imposed.
- `fittingStages` (list of JSON objects, default is `[]`): This setting instructs the optimizer to explore variations of the binding mode using a sequence of fitting stages. Each fitting stage can use a different set of variations and is defined by a JSON object that maps the included variations to `true`. The variations are:

- `optimizeSize`
- `optimizeSizeHeuristic`
- `optimizeFlankLength`
- `optimizeMotifShift`
- `optimizeMotifShiftHeuristic`

**`symmetry`** Function that defines the symmetry status of a binding mode

- `index` (integer, required): Specifies the index of symmetric binding mode
- `symmetryString` (string): Specifies the symmetry using the same format as in `bindingModeConstraints`

***Interactions***

**`addInteraction`** Function that adds interactions between binding modes

- `bindingModes` (list containing two integers, required): Indices of the interacting binding modes
- `positionBias` (boolean, default is `false`): If `true`, the binding mode interaction parameters $\omega_a(x,y)$ have independent values for each value of the binding mode configurations $x$ and $y$. If `false`, the interactions are translationally invariant and only depends on $x-y$ (where $x$ and $y$ are strand-aware coordinates).
- `maxOverlap` (integer, `0`): Maximum binding mode overlap
- `maxSpacing` (integer, default is `-1`): Maximum spacing between binding modes; `-1` indicates no limit

**`interactionConstraints`** Function that defines constraints on interaction parameters during optimization

- `index` (integer, required): Index of the constrained binding mode interaction
- `roundSpecificActivity` (boolean, default is `true`): Can interaction activities have independent values in different SELEX rounds (count table columns)?
- `experimentSpecificActivity` (boolean, default is `true`): Can interaction activities have independent values in different experiments (count tables)?
- `experimentSpecificInteraction` (boolean, default is `false`): Can interaction parameters have independent values in different experiments? This must be `true` if `positionBias=true` and the experiments have different probe lengths.

## Output

ProBound outputs the model in the form of a JSON Object with keys `modelSettings` and `coefficients`. Here, `modelSettings` specifies how the model was configured and `coefficients` contains all inferred parameter values. The latter object has the following keys:

- `countTable`: List of JSON Objects with the parameters for the count table models. Each object has the form:
  - `h`: List containing the values of $h_r \equiv \ln \eta_r$, where the index $r$ runs over rounds.
- `enrichmentModel`: List of JSON Objects with the parameters for the enrichment models. The only enrichment model with parameters is `rhoGamma`:
  - `rho`: List containing the values of $\rho_r$ where the index $r$ runs over rounds.
  - `gamma`: List containing the values of $\gamma_r$ where the index $r$ runs over rounds.

- `bindingModes`: List of JSON Objects with the parameters for the binding modes. Each object has the form:
  - `activity`: Two-level nested list containing the log-transformed binding mode activities $\ln \alpha_{e,r}$, where the indices $e$ runs over experiments (count tables) and $r$ runs over SELEX rounds (columns in the table).
  - `mononucleotide`: Single-level list containing the mononucleotide binding mode coefficients in $\vec{\beta}_a$ for binding mode $a$. This list can be thought of as a flattened PSAM: Letter $c$ at position $x$ in the PSAM has index $L*x+c$, where $L$ is the length of the alphabet. For the standard alphabet this corresponds to: $\{\beta_{A,1}, \beta_{C,1}, \beta_{G,1}, \beta_{T,1}, \beta_{A,2}...\}$.
  - `dinucleotide`: Two-level list containing the dinucleotide binding mode coefficients in $\vec{\beta}_a$ for binding mode $a$. The first index specifies the spacing between the interacting letters (0 is `NN`, 1 is `N.N`, etc). The second index can be thought of as a flattened dinucleotide PSAM: A dinucleotide feature with letters $c_1$ and $c_2$ and with the first letter on position $x$ has index $L^2x+Lc_1+c_2$, where $L$ is the length of the alphabet. For the standard alphabet this corresponds to $\{\beta_{AA,1}, \beta_{AC,1}, \beta_{AG,1}, \beta_{AT,1}, \beta_{CA,1}...\}$.
  - `positionBias`: Three-level list containing the position bias $\ln \omega(x)$. The indices are: (1) experiment, (2) stand, and (3) position in the sequence. The position is specified in the 5'-3' direction, meaning that the first position of the binding mode on the forward and reverse strands are on the opposite ends of the sequence.
- `bindingModeInteractions`: List of JSON Objects with the parameters for the binding mode interactions. Each object has the form:
  - `activity`: Two-level nested list containing the log-transformed binding mode interaction activities $\ln \alpha_{e,r}$, where the indices $e$ runs over experiments (count tables) and $r$ runs over SELEX rounds (columns in the table).
  - `positionMatrix`: Five-level list containing the binding mode interaction $\ln \omega(x,y)$. The indices are: (1) experiment, (2) stand of the first binding mode, (3) strand of the second binding mode, (4) position of the first binding mode in the sequence, and (5) position of the second binding mode in the sequence. The positions are specified in the 5'-3' direction, meaning that the first position of a binding mode on the forward and reverse strands are on the opposite ends of the sequence.

### ProBound configuration used in paper

ProBound was run with a variety of settings in order to learn the binding models shown in the figures. The corresponding configuration files are provided below. These settings utilize the two functions `addTableDB` and `oututDB` that only work in our internal computational environment, but both these functions can be substituted. For example,

```
{"function": "addTableDB", "count_table_id": 2600 }
```

loads a count table with internal count table ID `2600` using our database. This function call should be replaced with:

```
{"function": "addTable", "countTableFile": "UbxIVa-Hth-Exd.30mer1.tsv.gz",
        "inputFileType": "tsv.gz", "variableRegionLength":30,
        "nColumns": 4,
        "leftFlank": GTTCAGAGTTCTACAGTCCGACGATC,
        "rightFlank": CCCGGGTCGTATGCCGTCTTCTGCTTG }
```

The variable values for all count tables used below can be found in Extended Data Table S1 and S3. This table also contains the accession numbers for the published sequencing data used to generate the count tables (such as `UbxIVa-Hth-Exd.30mer1.tsv.gz`). The second internal function is

```
{"function": "outputDB", "fit_id": 6595 }
```

This function sets the ProBound output files using our internal database. This function call should be replaced with

```
{"function": "output", "outputPath": "/path/to/output", "baseName": "fit",
        "printTrajectory": true, "verbose": true }
```

This function directs the output to the directory `"/path/to/output"` and names of the output files start with `fit`. Finally, some of the settings below seed the binding mode to have the sequence readout at the center. The seeding strings were based on earlier unseeded fits that are not shown. These unseeded fits explored different sizes, shifts, and flank lengths of the binding modes using `optimizeFlankLength`, `optimizeMotifShiftHeuristic`, and `optimizeSizeHeuristic` as illustrated by the first setting below.

#### ***TF binding models, single-experiment***

In benchmarking ProBound, each training dataset was analyzed using three settings and the best binding model was then selected based on its ability to explain the training data (see Methods). The first setting utilized one non-specific binding mode (constant across sequences) and two PSAM binding modes. The size, frame shift and flank length of the PSAM binding modes were all optimized sequentially:

```
[
{"function": "optimizerSetting", "lambdaL2": 1e-6, "pseudocount": 20,
        "likelihoodThreshold": 0.0002 },
{"function": "addTableDB", "count_table_id": tableId },
{"function": "addSELEX" },
{"function": "addNS" },
{"function": "addBindingMode", "size": 12, "flankLength": 5},
{"function": "addBindingMode", "size": 12, "flankLength": 5},
{"function": "bindingModeConstraints", "index": 1, "maxFlankLength": -1,
        "maxSize": 18, "fittingStages": [
                { "optimizeFlankLength": true          },
                { "optimizeMotifShiftHeuristic": true },
                { "optimizeSizeHeuristic": true       } ] },
{"function": "bindingModeConstraints", "index": 2, "maxFlankLength": -1,
        "maxSize": 18, "fittingStages": [
                { "optimizeFlankLength": true          },
                { "optimizeMotifShiftHeuristic": true },
                { "optimizeSizeHeuristic": true       } ] },
{"function": "outputDB", "fit_id": fitID }
]
```

Here metadata for each count table (`variableRegionLength`, `nColumns`, `leftFlank`, `rightFlank`, and, when available, data accession numbers) is available in Extended Data Table S1. The second binding setting was equivalent to the first except for two changes: the non-specific binding mode was replaced by a 1bp PSAM that can absorb some sequence bias, and only the first and lasts available SELEX round was used:

```
[
{"function": "optimizerSetting", "lambdaL2": 1e-6, "pseudocount": 20,
        "likelihoodThreshold": 0.0002 },
{"function": "addTableDB", "count_table_id": tableID,
        "modeledColumns": [rFirst, rLast] },
{"function": "addSELEX"},
{"function": "addBindingMode", "size": 1, "singleStrand": true,
        "positionBias": true},
```

```
{"function": "addBindingMode", "size": 12, "flankLength": 5},
{"function": "addBindingMode", "size": 12, "flankLength": 5},
{"function": "bindingModeConstraints", "index": 1, "maxFlankLength": -1,
        "maxSize": 18, "fittingStages": [
                 { "optimizeFlankLength": true          },
                 { "optimizeMotifShiftHeuristic": true },
                 { "optimizeSizeHeuristic": true       } ] },
{"function": "bindingModeConstraints", "index": 2, "maxFlankLength": -1,
        "maxSize": 18, "fittingStages": [
                 { "optimizeFlankLength": true          },
                 { "optimizeMotifShiftHeuristic": true },
                 { "optimizeSizeHeuristic": true       } ] },
{"function": "outputDB", "fit_id": fitID }
]
```

Here `rFirst` and `rLast` should be replaced with the zero-based index of the first and last available SELEX round. The third setting was also identical to the first except it learned three PSAM binding modes:

```
[
{"function": "optimizerSetting", "lambdaL2": 1e-6, "pseudocount": 20,
        "likelihoodThreshold": 0.0002 },
{"function": "addTableDB", "count_table_id": tableID },
{"function": "addSELEX" },
{"function": "addNS" },
{"function": "addBindingMode", "size": 6, "flankLength": 5},
{"function": "addBindingMode", "size": 6, "flankLength": 5},
{"function": "addBindingMode", "size": 6, "flankLength": 5},
{"function": "bindingModeConstraints", "index": 1, "maxFlankLength": -1,
        "maxSize": 14, "fittingStages": [
                 { "optimizeFlankLength": true          },
                 { "optimizeMotifShiftHeuristic": true },
                 { "optimizeSizeHeuristic": true       } ] },
{"function": "bindingModeConstraints", "index": 2, "maxFlankLength": -1,
        "maxSize": 14, "fittingStages": [
                 { "optimizeFlankLength": true          },
                 { "optimizeMotifShiftHeuristic": true },
                 { "optimizeSizeHeuristic": true       } ] },
{"function": "bindingModeConstraints", "index": 3, "maxFlankLength": -1,
        "maxSize": 14, "fittingStages": [
                 { "optimizeFlankLength": true          },
                 { "optimizeMotifShiftHeuristic": true },
                 { "optimizeSizeHeuristic": true       } ] },
{"function": "outputDB", "fit_id": fitID }
]
```

***TF binding models, multiple experiments***

To learn learn a unified TF binding model from multiple SELEX datasets, the above three settings were modified to load and model multiple count tables. For example, the first setting was changed to be

```
[
{"function": "optimizerSetting", "lambdaL2": 1e-6, "pseudocount": 20,
        "likelihoodThreshold": 0.0002, "nThreads": 20 },
{"function": "addTableDB", "count_table_id": tableId1 },
{"function": "addTableDB", "count_table_id": tableId2 },
```

```
...
{"function": "addSELEX" },
{"function": "addSELEX" },
...
{"function": "addNS" },
{"function": "addBindingMode", "size": 12, "flankLength": 5 },
{"function": "addBindingMode", "size": 12, "flankLength": 5 },
{"function": "bindingModeConstraints", "index": 1, "maxFlankLength": -1,
        "maxSize": 18, "fittingStages": [
                { "optimizeFlankLength": true          },
                { "optimizeMotifShiftHeuristic": true },
                { "optimizeSizeHeuristic": true       } ] },
 {"function": "bindingModeConstraints", "index": 2, "maxFlankLength": -1,
        "maxSize": 18, "fittingStages": [
                { "optimizeFlankLength": true          },
                { "optimizeMotifShiftHeuristic": true },
                { "optimizeSizeHeuristic": true       } ]},
{"function": "outputDB", "fit_id": fitID }
]
```

Here one call to `addSELEX` is added each count table loaded using `addTableDB`.

#### *Combinatorial SELEX*

The Hth-Exd-Ubx CombSELEX-seq experiment was analyzed using following settings:

```
[
{"function": "optimizerSetting", "nThreads": 20, "lambdaL2": 1e-6,
        "pseudocount": 0, "likelihoodThreshold": 0.0002 },
{"function": "lbfgsSettings", "maxIters": 1000},
{"function": "addSELEXTableDB", "count_table_id": 2600,
        "bindingModes": [0, 1, 2, 3, 4 ],
        "bindingModeInteractions": [-1] },
{"function": "addSELEXTableDB", "count_table_id": 2703,
        "bindingModes": [0, 1, 2, 3    ],
        "bindingModeInteractions": [] },
{"function": "addSELEXTableDB", "count_table_id": 2702,
        "bindingModes": [0,       3    ],
        "bindingModeInteractions": [] },
{"function": "addSELEXTableDB", "count_table_id": 5653,
        "bindingModes": [0,    2       ],
        "bindingModeInteractions": [] },
{"function": "addSELEXTableDB", "count_table_id": 2680,
        "bindingModes": [0,          4 ],
        "bindingModeInteractions": [] },
{"function": "addNS" },
{"function": "addBindingMode", "size": 13,   "flankLength": 7,
        "dinucleotideDistance": 1 },
{"function": "addBindingMode", "size": 8,    "flankLength": 5,
        "dinucleotideDistance": 1 },
{"function": "addBindingMode", "size": 8,    "flankLength": 5,
        "dinucleotideDistance": 1 },
{"function": "addBindingMode", "size": 8,    "flankLength": 5,
        "dinucleotideDistance": 1 },
{"function": "bindingModeSeed", "index": 1,
```

```
        "mononucleotideIUPAC": "NATGATTTATGAN" },
{"function": "bindingModeSeed", "index": 2,
        "mononucleotideIUPAC": "NTTATGGN"    },
{"function": "bindingModeSeed", "index": 3,
        "mononucleotideIUPAC": "NTTGAYRN"    },
{"function": "bindingModeSeed", "index": 4,
        "mononucleotideIUPAC": "NNTGAYRN"    },
{"function": "addInteraction", "bindingModes": [1,4],
        "positionBias": false, "maxOverlap": 10 },
{"function": "interactionConstraints", "index": 0,
        "experimentSpecificInteraction": true },
{"function": "outputDB", "fit_id": 19837 }
]
```

Here each SELEX enrichment model is configured to included the appropriate biding modes and interactions, as indicated in Figure S2a, The interaction corresponds to the Hth-Exd-Ubx complex. An initial unseeded fit (not shown) was used to determine consensus sequence for each TF/complex, but some modes had unfavorable offsets in the PSAMs. In the final fit (above), the PSAMs were therefore seeded to have the sequence recognition in the center.

### *CAP-SELEX*

The matched CAP-SELEX and HT-SELEX experiments were analyzed using the following settings:

```
[
{"function": "addSELEXTableDB", "count_table_id": CAP-SELEX-table,
        "bindingModes": [0, 1, 2],
        "bindingModeInteractions": [-1] },
{"function": "addSELEXTableDB", "count_table_id": HT-SELEX-table-1,
        "bindingModes": [0, 1   ],
        "bindingModeInteractions": [] },
{"function": "addSELEXTableDB", "count_table_id": HT-SELEX-table-2,
        "bindingModes": [0,    2],
        "bindingModeInteractions": [] },
{"function": "optimizerSetting", "nThreads": 20, "lambdaL2": 1e-6,
        "pseudocount": 20, "likelihoodThreshold": 0.0002,
        "maxIters": 1000 },
{"function": "addNS" },
{"function": "addBindingMode", "size": 9, "flankLength": 4 },
{"function": "addBindingMode", "size": 10, "flankLength": 5 },
{"function": "bindingModeConstraints", "index": 1,
        "fittingStages": [ { "optimizeFlankLength": true } ],
        "maxFlankLength": -1},
{"function": "bindingModeConstraints", "index": 2,
        "fittingStages": [ { "optimizeFlankLength": true } ],
        "maxFlankLength": -1},
{"function": "bindingModeSeed", "index": 1,
        "mononucleotideIUPAC": "NNTGACANN"},
{"function": "bindingModeSeed", "index": 2,
        "mononucleotideIUPAC": "NNYAATTANN"},
{"function": "addInteraction", "bindingModes": [1,2],
        "positionBias": false,
        "maxOverlap": 5, "maxSpacing": 15 },
{"function": "interactionConstraints", "index": 0,
        "experimentSpecificInteraction": true },
```

```
{"function": "outputDB", "fit_id": 19849 }
]
```

***meCpG EpiSELEX-seq for ATF4 and CEBPγ***

The meCpG EpiSELEX-seq data for ATF4/CEBPγ was analyzed using the following settings:

```
[
{"function": "optimizerSetting", "nThreads": 20, "lambdaL2": 1e-6,
        "pseudocount": 0, "likelihoodThreshold": 0.0002 },
{"function": "addTableDB", "count_table_id": 3246,
        "transliterate": { "in": [],     "out": []    } },
{"function": "addTableDB", "count_table_id": 3247,
        "transliterate": { "in": ["CG"], "out": ["cg"]} },
{"function": "addTableDB", "count_table_id": 3218,
        "transliterate": { "in": [],     "out": []    } },
{"function": "addTableDB", "count_table_id": 3219,
        "transliterate": { "in": ["CG"], "out": ["cg"]} },
{"function": "addTableDB", "count_table_id": 3224,
        "transliterate": { "in": [],     "out": []    } },
{"function": "addTableDB", "count_table_id": 3225,
        "transliterate": { "in": ["CG"], "out": ["cg"]} },
{"function": "addSELEX", "bindingModes": [0, 1       ] },
{"function": "addSELEX", "bindingModes": [0, 1       ] },
{"function": "addSELEX", "bindingModes": [0,    2    ] },
{"function": "addSELEX", "bindingModes": [0,    2    ] },
{"function": "addSELEX", "bindingModes": [0, 1, 2, 3] },
{"function": "addSELEX", "bindingModes": [0, 1, 2, 3] },
{"function": "setAlphabet", "letterComplement": "C-G,A-T,c-g",
        "letterOrder": "ACGTcg" },
{"function": "addNS" },
{"function": "addBindingMode", "size": 12, "flankLength": 3,
        "dinucleotideDistance": 1 },
{"function": "addBindingMode", "size": 12, "flankLength": 3,
        "dinucleotideDistance": 1 },
{"function": "addBindingMode", "size": 12,  "flankLength": 3,
        "dinucleotideDistance": 1 },
{"function": "bindingModeSeed", "index": 1,
        "mononucleotideIUPAC": "NNTTGCATCANN" },
{"function": "bindingModeSeed", "index": 2,
        "mononucleotideIUPAC": "NNTGACGTCANN"       },
{"function": "bindingModeSeed", "index": 3,
        "mononucleotideIUPAC": "NNTTGCGCAANN"      },
{"function": "symmetry", "index": 2, "symmetryString": "1:12:1" },
{"function": "symmetry", "index": 3, "symmetryString": "1:12:1" },
{"function": "bindingModeConstraints", "index": 1,
        "fittingStages": [ { "optimizeFlankLength": true } ],
        "maxFlankLength": -1 },
{"function": "bindingModeConstraints", "index": 2,
        "fittingStages": [ { "optimizeFlankLength": true } ],
        "maxFlankLength": -1 },
{"function": "bindingModeConstraints", "index": 3,
        "fittingStages": [ { "optimizeFlankLength": true } ],
        "maxFlankLength": -1 },
```

```
{"function": "outputDB", "fit_id": 19831 }
]
```

Here, only the appropriate binding modes are included in each experiment (as indicated in Figure S4b) and `CG` is transliterated to `cg` in the modified libraries to encode meCpG. The PSAMs were seeded (based on an earlier unseeded fit) to have the sequence recognition at the center, and the homodimer binding modes were constrained to be reverse-complement symmetric.

### *meCpG, 5hmC and 6mA EpiSELEX-seq for CEBPγ*

The meCpG-, 5hmC-, and 6mA-aware binding model for CEBPγ was learned using the following settings:

```
[
{"function": "optimizerSetting", "nThreads": 20, "lambdaL2": 1e-6,
        "pseudocount": 0, "likelihoodThreshold": 0.0002 },
{"function": "addTableDB", "count_table_id": 3224,
        "transliterate": { "in": [],     "out": []    } },
{"function": "addTableDB", "count_table_id": 3225,
        "transliterate": { "in": ["CG"], "out": ["dh"]} },
{"function": "addTableDB", "count_table_id": 3227,
        "transliterate": { "in": ["C"],  "out": ["c"] } },
{"function": "addTableDB", "count_table_id": 3226,
        "transliterate": { "in": ["A"],  "out": ["a"] } },
{"function": "addSELEX" },
{"function": "addSELEX" },
{"function": "addSELEX" },
{"function": "addSELEX" },
{"function": "setAlphabet", "letterComplement": "C-G,A-T,a-t,c-g,d-h",
        "letterOrder": "ACGTacgtdh" },
{"function": "outputDB", "fit_id": 19843 },
{"function": "addNS" },
{"function": "addBindingMode", "size": 12, "flankLength": 3,
        "dinucleotideDistance": 1 },
{"function": "bindingModeSeed", "index": 1,
        "mononucleotideIUPAC": "NNTTGCGCAANN"},
{"function": "bindingModeConstraints", "index": 1,
        "fittingStages": [ { "optimizeFlankLength": true } ],
        "maxFlankLength": -1 },
{"function": "symmetry", "index": 1, "symmetryString": "1:12:1"}
]
```

These settings encode meCpG as `dh`, 5hmC:G as `c` (`g` on the reverse strand), and 6mA:T as `a` (`t` on the reverse strand). While this encoding differs from that displayed in Figure S4a, it is straightforward to update the encoding of the binding model.

### *RNA-binding proteins*

The RNA Bind-N-seq data for RBFOX2 was analyzed using the following settings:

```
[
{"function": "optimizerSetting", "nThreads": 20, "lambdaL2": 1e-6,
        "pseudocount": 200 },
{"function": "lbfgsSettings", "maxIters": 1000 },
{"function": "addTableDB", "count_table_id": 2479 },
{"function": "addTableDB", "count_table_id": 2483 },
{"function": "addTableDB", "count_table_id": 2478 },
```

```
{"function": "addTableDB", "count_table_id": 2482 },
{"function": "addTableDB", "count_table_id": 2477 },
{"function": "addTableDB", "count_table_id": 2481 },
{"function": "addTableDB", "count_table_id": 2476 },
{"function": "addTableDB", "count_table_id": 2480 },
{"function": "addTableDB", "count_table_id": 2484 },
{"function": "addSELEX", "bindingSaturation": true,
        "concentration": 1   },
{"function": "addSELEX", "bindingSaturation": true,
        "concentration": 4   },
{"function": "addSELEX", "bindingSaturation": true,
        "concentration": 14  },
{"function": "addSELEX", "bindingSaturation": true,
        "concentration": 40  },
{"function": "addSELEX", "bindingSaturation": true,
        "concentration": 121 },
{"function": "addSELEX", "bindingSaturation": true,
        "concentration": 365 },
{"function": "addSELEX", "bindingSaturation": true,
        "concentration": 1100},
{"function": "addSELEX", "bindingSaturation": true,
        "concentration": 3300},
{"function": "addSELEX", "bindingSaturation": true,
        "concentration": 9800},
{"function": "addNS" },
{"function": "addBindingMode", "size": 10, "flankLength": 6,
        "singleStrand": true, "dinucleotideDistance": 10 },
{"function": "bindingModeConstraints", "index": 1,
        "roundSpecificActivity": false,
        "experimentSpecificActivity": false },
{"function": "bindingModeSeed", "index": 1,
        "mononucleotideString": "..TGCATG.."},
{"function": "outputDB", "fit_id": 16567 }
]
```

Here the SELEX model constrained the experiment-specific activities to be proportional to the RBP concentrations used in each experiment, and the binding mode was configured include all-by-all interactions and to only score the forward strand. The 1nM, 4nM and 14nM experiments have very weak binding enrichment and are not shown in Figure S7f.

### $K_D$-seq - single experiment

The single-concentration $K_\mathrm{D}$ analyses used the following configuration:

```
[
{ "function": "optimizerSetting", "lambdaL2": 1e-6, "nThreads": 20,
        "pseudocount": 200 },
{ "function": "lbfgsSettings", "maxIters": 1000},
{ "function": "addTableDB", "count_table_id": 5137 },
{ "function": "addSELEX", "modelType": "RhoGamma", "concentration": 100,
        "cumulativeEnrichment": false },
{ "function": "addNS" },
{ "function": "addBindingMode", "size": 10, "flankLength": 6,
        "dinucleotideDistance": 10 },
{ "function": "bindingModeConstraints", "index": 0,
```

```
        "roundSpecificActivity": false },
{ "function": "bindingModeConstraints", "index": 1,
        "roundSpecificActivity": false },
{ "function": "enrichmentModelSeed", "index": 0, "rho": [0,1,0],
        "gamma": [0,-1,-1] },
{ "function": "bindingModeSeed", "index": 1,
        "mononucleotideString": "..TAATTG.." },
{ "function": "outputDB", "fit_id": 16609 }
]
```

### ***$K_D$-seq - multiple experiments***

The multi-concentration $K_\mathrm{D}$ analyses of the Input/Bound/Free libraries used the following configuration:

```
[
{ "function": "optimizerSetting", "lambdaL2": 1e-6, "nThreads": 20,
    "pseudocount": 1000 },
{ "function": "addTableDB", "count_table_id": 5134 },
{ "function": "addTableDB", "count_table_id": 5135 },
{ "function": "addTableDB", "count_table_id": 5136 },
{ "function": "addTableDB", "count_table_id": 5137 },
{ "function": "addSELEX", "modelType": "RhoGamma", "concentration": 3300,
        "cumulativeEnrichment": false },
{ "function": "addSELEX", "modelType": "RhoGamma", "concentration": 1000,
        "cumulativeEnrichment": false },
{ "function": "addSELEX", "modelType": "RhoGamma", "concentration": 330,
        "cumulativeEnrichment": false },
{ "function": "addSELEX", "modelType": "RhoGamma", "concentration": 100,
        "cumulativeEnrichment": false },
{ "function": "addNS" },
{ "function": "addBindingMode", "size": 10, "flankLength": 6,
        "dinucleotideDistance": 10 },
{ "function": "bindingModeConstraints", "index": 0,
        "roundSpecificActivity": false,
        "experimentSpecificActivity": false },
{ "function": "bindingModeConstraints", "index": 1,
        "roundSpecificActivity": false,
        "experimentSpecificActivity": false },
{ "function": "enrichmentModelSeed", "rho": [0,1,0],
        "gamma": [0,-1,-1] },
{ "function": "bindingModeSeed", "index": 1,
        "mononucleotideString": "..TAATTG.."},
{ "function": "outputDB", "fit_id": 19357 }
]
```

The analyses that instead analyzed the Input/Bound and Bound/Free libraries used the same configuration but with the arguments `"modeledColumns": [0,1]` and `"modeledColumns": [1,2]`, respectively, added to `addTable`.

### ***Peak-free ChIP-seq motif discovery - single experiment***

The binding models for GR and its cofactors were learned from ChIP-seq data using the following settings:

```
[
{ "function": "optimizerSetting", "nThreads": 20, "lambdaL2": 1e-6,
        "pseudocount": 20, "likelihoodThreshold": 0.0002 },
```

```
{ "function": "addTableDB", "count_table_id": 4974 },
{ "function": "addSELEX", "modelType": "SELEX",
        "cumulativeEnrichment": true },
{ "function": "addNS" },
{ "function": "addBindingMode", "size": 15, "flankLength": 0,
        "dinucleotideDistance": 0, "positionBias": true },
{ "function": "addBindingMode", "size": 10, "flankLength": 0,
        "dinucleotideDistance": 0, "positionBias": true },
{ "function": "addBindingMode", "size": 10, "flankLength": 0,
        "dinucleotideDistance": 0, "positionBias": true },
{ "function": "addBindingMode", "size": 10, "flankLength": 0,
        "dinucleotideDistance": 0, "positionBias": true },
{ "function": "bindingModeSeed"       , "index": 1,
        "mononucleotideString": "AG.ACA...TGT.CT" },
{ "function": "symmetry",               "index": 1,
        "symmetryString": "abcdefg1GFEDCBA" },
{ "function": "bindingModeConstraints",
        "index": 1, "positionBiasBinWidth": 5 },
{ "function": "bindingModeConstraints", "index": 2,
        "fittingStages": [
                { "optimizeMotifShiftHeuristic": true },
                { "optimizeSize": true } ],
        "maxSize": 18, "positionBiasBinWidth": 5 },
{ "function": "bindingModeConstraints", "index": 3,
        "fittingStages": [
                { "optimizeMotifShiftHeuristic": true },
                { "optimizeSize": true } ],
        "maxSize": 18, "positionBiasBinWidth": 5 },
{ "function": "bindingModeConstraints", "index": 4,
        "fittingStages": [
                { "optimizeMotifShiftHeuristic": true },
                { "optimizeSize": true } ],
        "maxSize": 18, "positionBiasBinWidth": 5 },
{ "function": "outputDB", "fit_id": 19732 }
]
```

Here the GR binding mode was configured to be reverse-complement symmetric.

***Peak-free ChIP-seq motif discovery - multiple agonist treatments***

The impact of CORT treatment GR binding was quantified using the following settings:

```
[
{ "function": "optimizerSetting", "lambdaL2": 1e-6,
        "pseudocount": 20, "likelihoodThreshold": 0.0002 },
{"function": "addTableDB", "count_table_id": 4873 },
{"function": "addTableDB", "count_table_id": 4874 },
{"function": "addTableDB", "count_table_id": 4875 },
{"function": "addSELEX" },
{"function": "addSELEX" },
{"function": "addSELEX" },
{ "function": "addNS" },
{ "function": "addBindingMode", "size": 15, "flankLength": 0,
        "dinucleotideDistance": 0 },
{ "function": "bindingModeConstraints", "index": 1,
```

```
        "roundSpecificActivity": true,
        "experimentSpecificActivity": true },
{ "function": "bindingModeSeed", "index": 1,
        "mononucleotideString": "AG.ACA...TGT.CT" },
{ "function": "symmetry", "index": 1,
        "symmetryString": "abcdefg1GFEDCBA" },
{ "function": "outputDB", "fit_id": 19733 }
]
```

Here the binding mode is configured to have independent activities in each experiment.

***Kinase sequence specificity***

The peptide-sequence specificity of tyrosine kinase Src was quantified using the following settings:

```
[
{"function": "optimizerSetting", "nThreads": 20, "lambdaL2": 1e-6,
        "pseudocount": 50 },
{"function": "lbfgsSettings", "maxIters": 2000 },
{"function": "addTableDBs", "count_table_ids": [4831,4830,4832] },
{"function": "addSELEX", "modelType": "ExponentialKinetics",
        "concentration": 0.25 },
{"function": "addSELEX", "modelType": "ExponentialKinetics",
        "concentration": 1    },
{"function": "addSELEX", "modelType": "ExponentialKinetics",
        "concentration": 3    },
{"function": "setAlphabet", "letterComplement":
        "A-A,C-C,D-D,E-E,F-F,G-G,H-H,I-I,K-K,L-L,M-M,N-N,P-P,Q-Q, \\
        R-R,S-S,T-T,V-V,W-W,Y-Y",
        "letterOrder": "ACDEFGHIKLMNPQRSTVWY" },
{"function": "addNS" },
{"function": "addBindingMode",  "size": 7, "flankLength": 3,
        "singleStrand": true, "dinucleotideDistance": 7},
{"function": "bindingModeConstraints", "index": 1,
        "experimentSpecificActivity": false },
{"function": "symmetry", "index": 1, "symmetryString": "abc.efg" },
{"function": "bindingModeSeed", "index": 1,
        "mononucleotideString": "...Y...",
        "seedScale": 10 },
{"function": "enrichmentModelConstraints", "index": -1,
        "fitDelta": [false, false]},
{"function": "enrichmentModelSeed", "index": -1, "delta": [0,-15] },
{"function": "outputDB", "fit_id": 16581 }
]
```

Here the `concentration` setting was used to encode the different exposures of the experiments (5min, 20min and 60min were encodes as 0.25, 1, and 3) and an extended and self-complementary alphabet was used to represent peptides. The binding mode was configured to include all-by-all interactions between the peptides and only the forward strand was scored. The commands `bindingModeSeed` and `symmetry` were used to fix the central position to recognize `Y`.
