# Supplementary material for: Prediction of protein–ligand binding affinity from sequencing data with interpretable machine learning
Source: Nat Biotechnol. 2022 May 23;40(10):1520–7. doi: 10.1038/s41587-022-01307-0 (PMC9546773; doi:10.1038/s41587-022-01307-0)
Supplement: Supplementary file 2 — Reporting Summary [file 41587_2022_1307_MOESM2_ESM.pdf]

nature research

Corresponding author(s): Harmen J. Bussemaker

Last updated by author(s): Mar 4, 2022

Reporting Summary

Nature Research wishes to improve the reproducibility of the work that we publish. This form provides structure for consistency and transparency in reporting. For further information on Nature Research policies, see our Editorial Policies and the Editorial Policy Checklist.

Statistics

For all statistical analyses, confirm that the following items are present in the figure legend, table legend, main text, or Methods section.

n/a	Confirmed	
☐	☒	The exact sample size (*n*) for each experimental group/condition, given as a discrete number and unit of measurement
☐	☒	A statement on whether measurements were taken from distinct samples or whether the same sample was measured repeatedly
☐	☒	The statistical test(s) used AND whether they are one- or two-sided<br>*Only common tests should be described solely by name; describe more complex techniques in the Methods section.*
☐	☒	A description of all covariates tested
☒	☐	A description of any assumptions or corrections, such as tests of normality and adjustment for multiple comparisons
☐	☒	A full description of the statistical parameters including central tendency (e.g. means) or other basic estimates (e.g. regression coefficient) AND variation (e.g. standard deviation) or associated estimates of uncertainty (e.g. confidence intervals)
☐	☒	For null hypothesis testing, the test statistic (e.g. *F*, *t*, *r*) with confidence intervals, effect sizes, degrees of freedom and *P* value noted<br>*Give P values as exact values whenever suitable.*
☒	☐	For Bayesian analysis, information on the choice of priors and Markov chain Monte Carlo settings
☒	☐	For hierarchical and complex designs, identification of the appropriate level for tests and full reporting of outcomes
☐	☒	Estimates of effect sizes (e.g. Cohen's *d*, Pearson's *r*), indicating how they were calculated

Our web collection on statistics for biologists contains articles on many of the points above.

Software and code

Policy information about availability of computer code

Data collection	EMSA gels were quantitated using FIJI (v. 1.52n). HPLC data were quantitated using Agilent OpenLabs ChemStation (v. C.01.09). Paired-end reads were merged using FLASH (v. FLASH2-2.2.00). Adapter sequences were trimmed using Cutadapt (v3.5).
Data analysis	Massively parallel sequencing data were analyzed using custom-written software as described in the Methods section.

For manuscripts utilizing custom algorithms or software that are central to the research but not yet described in published literature, software must be made available to editors and reviewers. We strongly encourage code deposition in a community repository (e.g. GitHub). See the Nature Research guidelines for submitting code & software for further information.

Data

Policy information about availability of data

All manuscripts must include a data availability statement. This statement should provide the following information, where applicable:

- Accession codes, unique identifiers, or web links for publicly available datasets
- A list of figures that have associated raw data
- A description of any restrictions on data availability

The sequencing data generated during the current study have been deposited in the Gene Expression Omnibus (GEO, accession number GSE175942). Source data for Figs 4d and 6d have been provided in Supplemental Table S2 and S5.

Field-specific reporting

Please select the one below that is the best fit for your research. If you are not sure, read the appropriate sections before making your selection.

☒ Life sciences ☐ Behavioural & social sciences ☐ Ecological, evolutionary & environmental sciences

For a reference copy of the document with all sections, see nature.com/documents/nr-reporting-summary-flat.pdf

Life sciences study design

All studies must disclose on these points even when the disclosure is negative.

Sample size	The conclusions were based on high-throughput in vitro assays yielding millions of data points each, and typically one replicate per assay.
Data exclusions	No data were excluded.
Replication	The robustness of the novel K_D-seq assay was established by repeating the assay over all combinations of three DNA and four protein concentrations, as discussed in depth in the main text. Each dataset was included.
Randomization	The in vitro selection assays described in the study do not require the use of a traditional randomized design to address confounding. Instead, information is obtained by analyzing the internal variation within each dataset.
Blinding	The low-throughput data used to validate the models were generated by different operators than those who generated the models, and the former had no access to the models and the predictions made using the models when they generated the validation measurements.

Reporting for specific materials, systems and methods

We require information from authors about some types of materials, experimental systems and methods used in many studies. Here, indicate whether each material, system or method listed is relevant to your study. If you are not sure if a list item applies to your research, read the appropriate section before selecting a response.

Materials & experimental systems

n/a	Involved in the study	
☐	☒	Antibodies
☒	☐	Eukaryotic cell lines
☒	☐	Palaeontology and archaeology
☒	☐	Animals and other organisms
☒	☐	Human research participants
☒	☐	Clinical data
☒	☐	Dual use research of concern

Methods

n/a	Involved in the study	
☒	☐	ChIP-seq
☒	☐	Flow cytometry
☒	☐	MRI-based neuroimaging

Antibodies

Antibodies used	The phosphotyrosine monoclonal antibody (pY20, conjugated to the fluorophore, perCP-eFluor 710, Invitrogen, catalog 46-5001-42), was desthiobiotinylated before use in the specificity screen.
Validation	This is a well-established mouse monoclonal pan-phosphotyrosine antibody, which was purchased from Invitrogen. The vendor provides flow cytometry validation data on stimulated vs. unstimulated lymphocytes (fixed and permeabilized for intracellular staining). The validation data clearly show a marked increase in phosphotyrosine levels upon stimulation, consistent with known lymphocyte signaling. We have also validated the specificity of this antibody in our bacterial surface-display setup. Cells expressing displayed peptides, treated with a tyrosine kinase without ATP show no background antibody staining. By contrast, cells expressing displayed peptides, treated with tyrosine kinase and 1mM ATP show increasing antibody staining as a function of phosphorylation time. Note that for this study, we modified this commercial antibody with desthiobiotin using the DSB-X labeling kit from Molecular Probes (catalog # D20655), following the manufacturer's protocol.
